# Heterologous vaccination utilizing viral vector and protein platforms confers complete protection against SFTSV

**DOI:** 10.1038/s41598-023-35328-9

**Published:** 2023-05-20

**Authors:** Jae-Yong Kim, Kyeongseok Jeon, Jung Joo Hong, Sang-In Park, Hyeonggon Cho, Hyo-Jung Park, Hye Won Kwak, Hyeong-Jun Park, Yoo-Jin Bang, Yu-Sun Lee, Seo-Hyeon Bae, So-Hee Kim, Kyung-Ah Hwang, Dae-Im Jung, Seong Hoo Cho, Sang Hwan Seo, Green Kim, Hanseul Oh, Hwal-Yong Lee, Ki Hyun Kim, Hee-Young Lim, Pyeonghwa Jeon, Joo-Yeon Lee, Junho Chung, Sang-Myeong Lee, Hae Li Ko, Manki Song, Nam-Hyuk Cho, Young-suk Lee, So-Hee Hong, Jae-Hwan Nam

**Affiliations:** 1 Department of Medical and Biological Sciences, The Catholic University of Korea, 43-1 Yeokgok-dong, Wonmi-gu, Bucheon, 14662 Republic of Korea; 2 BK Plus Department of Biotechnology, The Catholic University of Korea, Bucheon, Gyeonggi-do Republic of Korea; 3 SML Biopharm, Gwangmyeong, Gyeonggi-do Republic of Korea; 4 Department of Biomedical Sciences, Seoul National University College of Medicine, Seoul, 03080 Republic of Korea; 5 Immunology and Infectious Disease Lab, National Primate Research Center, Korea Research Institute of Bioscience and Biotechnology (KRIBB)/University of Science and Technology, 30 Yeongudanji-ro, Ochang-eup, Cheongwon-gu, Cheongju-si, Chungcheongbuk-do 28116 Republic of Korea; 6 Department of Bio and Brain Engineering, Korea Advanced Institute of Science and Technology (KAIST), Daejeon, 34141 Republic of Korea; 7 Department of Research and Development, Genetree Research, Seoul, Republic of Korea; 8 Science Unit, International Vaccine Institute, Seoul, Republic of Korea; 9 Department of Biochemistry and Molecular Biology, Seoul National University College of Medicine, Seoul, 03080 Republic of Korea; 10 Center for Emerging Virus Research, National Institutes of Health, Korea Disease Control and Prevention Agency, Cheongju, Republic of Korea; 11 College of Veterinary Medicine, Chungbuk National University, Cheongju, Republic of Korea; 12 Scripps Korea Antibody Institute, Chuncheon, 24341 Republic of Korea; 13 Department of Microbiology, College of Medicine, Ewha Womans University, Seoul, 07804 Republic of Korea

**Keywords:** Immunology, Vaccines

## Abstract

Severe fever with thrombocytopenia syndrome virus was first discovered in 2009 as the causative agent of severe fever with thrombocytopenia syndrome. Despite its potential threat to public health, no prophylactic vaccine is yet available. This study developed a heterologous prime-boost strategy comprising priming with recombinant replication-deficient human adenovirus type 5 (rAd5) expressing the surface glycoprotein, Gn, and boosting with Gn protein. This vaccination regimen induced balanced Th1/Th2 immune responses and resulted in potent humoral and T cell-mediated responses in mice. It elicited high neutralizing antibody titers in both mice and non-human primates. Transcriptome analysis revealed that rAd5 and Gn proteins induced adaptive and innate immune pathways, respectively. This study provides immunological and mechanistic insight into this heterologous regimen and paves the way for future strategies against emerging infectious diseases.

## Introduction

Severe fever with thrombocytopenia syndrome (SFTS) is an emerging tick-borne infectious disease that was discovered in Central China in 2009^1^. The causative agent, Dabie bandavirus, widely known as SFTS virus (SFTSV), is a single-stranded negative-sense RNA virus belonging to the genus *Bandavirus* of the family Phenuiviridae^2^. The SFTSV genome encodes three segments—namely, L, M, and S. The L segment encodes the RNA-dependent RNA polymerase (RdRp), whereas the M segment encodes the surface glycoproteins Gn and Gc. The Gn protein is a target protein for neutralizing antibodies. The S segment of the virus contains the genetic information for both nucleocapsid proteins (NP) and nonstructural proteins (NSs)^3,4^. Since the first reported case of SFTS in humans, the number of human cases has rapidly increased each year in China, South Korea, and Japan^5^. Furthermore, diseases that are caused by the *Phlebovirus* genus, which are highly similar to SFTS, have also emerged in Mediterranean countries and the United States^6,7^.

The typical symptoms of SFTS include high-grade fever, thrombocytopenia, leukocytopenia, and gastrointestinal disorders. Multi-organ failure and disseminated intravascular coagulation occur in severe cases. The fatality rate ranges from 5 to 30%, depending on the region surveyed and the amount of reporting in that area^8^. Due to the increase in SFTS cases from 2012 to 2018 and the severity of symptoms, the World Health Organization has included SFTSV in the list of priority target pathogens that require urgent attention^9^.

Since there is currently no vaccine available and considering the high mortality rate associated with the syndrome and the potential for future outbreaks, efforts to develop an effective vaccine are needed. To date, few research groups have attempted to develop vaccines against SFTS. A DNA vaccine encoding full-length Gn, Gc, N, NS, and RdRp genes of SFTSV had been reported to elicit T cell responses and neutralizing antibodies and protect against lethal SFTSV infection in a ferret model^10^. Kang et al.^11^ developed a single recombinant plasmid DNA encoding SFTSV genes, Gn and Gc together with NP-NS fusion antigen, as a vaccine candidate. They fused the four viral antigens with Fms-like tyrosine kinase-3 ligand (Flt3L) and interleukin (IL)-12 gene and incorporated them into the plasmid to enhance cell-mediated immunity. Although it failed to induce neutralizing antibodies; it provided complete protection against lethal SFTSV infection in interferon/receptor knockout (IFNAR KO) mice by inducing T-cell responses. In addition, a single dose of recombinant replication-deficient human adenovirus type 5 (Ad5) co-expressing rabies virus (RABV) glycoprotein and SFTSV Gn (Ad5-G-Gn) induced robust neutralizing antibodies against both SFTSV and RABV as well as a reduction in splenic viral loads after challenge^12^.

The vaccine candidates mentioned above have several drawbacks. Although DNA vaccines successfully protect against lethal challenges, safety concerns regarding DNA vaccines or their weak induction of neutralizing antibodies act as limitations^13,14^. Furthermore, it remains unclear whether a single dose-Ad5-G-Gn can provide protection against SFTSV challenge and induce a certain degree of neutralizing antibody responses. Protein subunit vaccines tend to induce humoral immune responses, but they usually elicit a low level of T cell-mediated immune responses^15^. The ssRNA adjuvant utilized in this study was derived from the intergenic region internal ribosome entry site of the cricket paralysis virus^15–17^. In our previous study, heterologous prime-boost vaccination regimens containing Ad5/MERS resulted in concurrent Th1 and Th2 responses, whereas homologous prime-boost regimens did not. Owing to Th1/Th2 balance, heterologous prime-boost vaccination induces enduring immune responses against MERS-CoV due to the appropriate balance of Th1/Th2 responses^18^. Recently, heterologous prime-boost, a novel vaccination method that uses different vaccine platforms, has been extensively researched, and many studies have confirmed that it is more effective than the traditional homologous prime-boost strategy^18–22^.

This study developed a heterologous prime-boost strategy by priming with adenovirus serotype 5 (rAd5), which contains the transmembrane-deleted Gn gene, and boosting with stem region deleted recombinant Gn protein formulated with RNA adjuvant to maximize both humoral and cellular immune responses. Furthermore, how the heterologous prime-boost strategy could synergistically upregulate immune responses was investigated.

## Materials and methods

### SFTS GnΔSTEM subunit protein production and purification

The insect cell codon-optimized gene for SFTS GnΔSTEM was synthesized (GenScript, Piscataway, NJ, USA). This synthetic GnΔSTEM gene has a *BamH*I site and a secretory signal sequence (Gp67) at 5′ end and, 6 × His-tag sequences and the *Sal*I site at 3′ end. The gene was cloned into the pFastBac donor vector (Thermo Fisher Scientific, Waltham, MA, USA) using *BamH*I and *Sal*I restriction sites. Infectious recombinant baculovirus was prepared as described previously^23^. For recombinant protein expression, *Spodoptera frugiperda* (Sf9) insect cells (Thermo Fisher Scientific) were transfected with the plasmid DNA encoding Gn∆STEM. At 3 days of transfection, the cell culture supernatant was harvested as the first passage (P1) of recombinant baculovirus. For the GnΔSTEM protein purification, P3 baculovirus was prepared, and Sf9 cells were infected with the P3 baculovirus. After 4 days of incubation at 27 °C in a non-CO_2_, non-humidified incubator with shaking (150 rpm), the cell culture supernatant was collected and filtered through a 0.2-μm filter (Nalgene™, Thermo Fisher Scientific). The filtered protein was purified using Akta start affinity chromatography (GE Healthcare, Uppsala, Sweden). The imidazole in the purified protein was removed using an Amicon tube (MWCO of 10 kDa, UFC901024, Millipore, Billerica, MA, USA), and the concentration and purity of the protein were measured using a bicinchoninic acid kit (Thermo Fisher Scientific) and sodium dodecyl sulfate–polyacrylamide gel electrophoresis (SDS-PAGE), respectively. Information on the sequence of baculovirus expression vector and preparation of infectious recombinant baculovirus is provided in the supplementary information.

### Generation of human adenovirus 5 expressing SFTSV GnΔTM

Recombinant adenoviruses encoding the SFTSV Gn protein were purchased from SIRION Biotech (Munich, Germany). Briefly, the production protocol is as follows: The CMV-GnΔTM region of the shuttle plasmid was transferred via recombination in a BAC vector containing the genome of a replication-deficient Ad5-based vector with deleted E1/E3 genes. After the release of recombinant viral DNA from the purified BAC-DNA by restriction digest with *Pac* I, HEK293 cells plated on the previous day in 6-well plates were transfected with adenoviral DNA and incubated for 3 days at 37 °C. The cells were subsequently harvested and subjected to a freeze/thaw treatment. Fresh HEK293 cells plated on the previous day in 6-well plates were then infected with the resultant cell lysate and incubated at 37 °C until the cells showed complete cytopathic effects. Afterward, the cells were harvested, and viral particles were released by freeze/thaw treatment. Infectious units (IU) were determined via immunohistochemical detection of the adenoviral hexon protein in HEK 293 cells infected with serial dilutions by SIRION Biotech.

### In vitro transcription

A DNA platform was designed using IGR IRES and SV40 late polyadenylation signal sequences^17^. DNA templates were linearized with NotI. In vitro transcription was performed using the EZ™ T7 high-yield in vitro transcription kit (Enzynomics, Daejeon, South Korea). For transcription using the Enzynomics Transcription kit, a 3 μg linearized DNA template was incubated with T7 transcription buffer, MgCl_2_, 10 mM DTT, enhancer solution, 5 mM rNTP, nuclease-free water, and 200 U T7 enzyme mix for 1 h at 37 °C. The transcripts were incubated with RNase-free DNase I (Enzynomics) for 30 min at 37 °C, followed by termination of the reaction by incubation at 65 °C for 10 min. RNA purification was performed using the LiCl method. RNA purification and concentration were evaluated using a NanoDrop-2000 spectrophotometer (Thermo Fisher Scientific, Waltham), and RNA integrity was analyzed through denaturing gel electrophoresis.

### Western blot analysis

For western blot analysis, 1 × 10^6^ of HEK293 cells were infected with 1 × 10^7^ infectious units of rAd5-GnΔTM or rAd5-GFP. The cells were incubated for 24 h, and cell lysates were separated using SDS-PAGE and transferred to a nitrocellulose membrane. The membrane was blocked with PBS containing 0.1% Tween-20 and 5% skim milk for 1 h at room temperature. After blocking, the membrane was incubated overnight at 4℃ with anti-SFTSV Gn antibody (1:1000 dilution, NBP2-41,156, Novus Biological, Centennial, CO, USA) and anti-beta actin antibody (1:3000 dilution, 3700S, Cell Signaling Technology, Danvers, MA, USA). Anti-rabbit or anti-mouse antibody conjugated with horse radish peroxidase (1:5000 dilution, A120-101P or A90-116P, BETHYL, Montgomery, TX, USA) was used as the secondary antibody. The signals were detected with an ECL substrate solution (ECL-PS100, Donginbio, Seoul, South Korea).

### Mice

Six-week-old female C57BL/6 mice were purchased from Samtako BioKorea (Osan, Republic of Korea) or Dae-Han Bio-Link (Chungbuk, Republic of Korea). Furthermore, 10-month-old female C57BL/6 mice were purchased from the same manufacturers and raised until 16 months old. Animals were housed at the Catholic University of Korea under specific pathogen-free conditions and a standard light cycle (12-h light/dark cycle). All experimental procedures conducted on animals in this study complied with the ARRIVE guidelines and followed the guidelines of and were approved by the Institutional Animal Care and Use Committee of the Catholic University of Korea (approval number: CUK-IACUC-2020-015, BA-2008-301-071-01). The animal facility was fully accredited by the Korean Association for Laboratory Animals (2018-027; August 24, 2018).

### Mice immunization and challenge

C57BL/6 WT mice were intramuscularly immunized twice at 2-week intervals with rAd5-Gn or alum-formulated Gn protein with CrPV mRNA adjuvant^15,17^. Alum and CrPV mRNA adjuvant were solely used with the Gn protein and not with rAd5-Gn. At 2 weeks after the final immunization, the mice were subcutaneously challenged with 1 × 10^5^ FFU of SFTSV^24^. Body weight and mouse survival were monitored until the surviving mice fully recovered.

### Preparation of SFTSV

SFTSV (GenBank accession no.: MN329148-MN329150) isolated from a Korean patient with SFTS was propagated in Vero E6 cells (ATCC CRL-1586). The supernatant of the infected cells was harvested five days after infection and stored at − 80 °C after filtering with a 0.45-μm syringe. The FFU of SFTSV was determined by focus forming assay using methylcellulose media^11^.

### Flow cytometry

Mouse splenocytes (1 × 10^6^) were stained with fluorochrome-conjugated antibodies combined with a solution of PBS and 0.5% fetal bovine serum for flow cytometry. To evaluate the follicular helper T cells, memory T cells, and T cell activation, isolated splenocytes were blocked using CD16/CD32 (14-0161-85, Invitrogen, Waltham, MA, USA) for 20 min at 4 ℃ and then stained with LIVE/DEAD dead cell stain kit (L34957, Invitrogen), anti-mouse CD4 (11-0041-82, Invitrogen), CXCR5 (145,517, BioLegend, San Diego, CA, USA), ICOS (25-9948-42, Thermo Fisher), PD-1 (135,255, BioLegend), CD44 (17-0441-82, Invitrogen), CD62L (104,410, BioLegend), CD25 (25-0251-82, Invitrogen), and CD69 (104,514, BioLegend) for 30 min at 4 ℃ in the dark. To stain intra-cellular cytokines, isolated splenocytes were stimulated with 1 μg/mL of Gn protein for 16 h at 37 °C, and brefeldin A (GolgiPlug, BD Biosciences, Franklin Lakes, NJ, USA) was added at 6 h post-stimulation. After another 10 h of incubation, splenocytes were blocked using CD16/CD32 (14-0161-85, Invitrogen) for 20 min at 4 °C and then stained with CD4 (100,414, BioLegend), CD8 (100,737, BioLegend), and LIVE/DEAD dead cell stain kit (L34957, Invitrogen) for 30 min at 4 °C in the dark. The stained cells were permeabilized using a Fixation/Permeabilization Solution kit (BD Biosciences) for 1 h at 4 °C in the dark and then stained with IFN-γ (505,802, BioLegend) and TNF-α (506,304, BioLegend) for 30 min at 4 °C in the dark. After washing, the cells were analyzed using a Cytek Aurora flow cytometer (Cytek, Fremont, CA, USA), and the results were interpreted using SpectroFlo software (Cytek). The gating strategies are depicted in Supplementary Fig. 5.

### Enzyme-linked immunosorbent spot (ELISpot) assays

To perform the ELISpot assay for mice, splenocytes of the immunized mice were stimulated with 1 μg/mL of Gn protein, which was used for immunization in this study, or 1 μg/mL of SFTSV peptide mixture for 48 h. IFN-γ-secreting cells were detected using the mouse IFN-γ ELISpot^basic^ kit (3321-4APT-10, Mabtech, Nacka Strand, Sweden), respectively. The manufacturer’s protocol was used for these assays.

### Enzyme-linked immunosorbent assay (ELISA)

Antigen-specific IgG1 and IgG2c levels in mouse serum or non-human primates (NHP) plasma were measured using ELISA. The 96-well plates (Corning, Corning, NY, USA) were coated with Gn protein (100 ng/well) at 4 °C overnight. The wells were then washed three times with 200 µL of PBS containing 0.05% Tween 20 (PBS-T) and blocked with 100 µL blocking buffer (PBS containing 1% bovine serum albumin) for 1 h at 18–25 °C. After adding diluted samples to the wells, the plates were incubated at room temperature for 2 h. After incubation, the wells were washed thrice with 200 µL of PBS-T. Horseradish peroxidase (HRP)-conjugated anti-mouse IgG1 (A90-105P, Bethyl), IgG2c (A90-136P, Bethyl), and anti-monkey IgG-HRP conjugated antibodies (617-103-012, Rockland Immunochemicals, Pottstown, PA, USA) diluted 1/10,000 in blocking buffer were added and incubated for 1 h at 18–25 °C. After five washes with PBS-T, tetramethylbenzidine substrate (Invitrogen, USA) was added and incubated for 5–10 min, and 2N H_2_SO_4_ was added to terminate the reaction. The optical density values were measured at 450 nm using a GloMax Explorer microplate reader (Promega, Madison, WI, USA).

### RT-qPCR

SFTS was confirmed by the detection of SFTSV RNA through RT-PCR analysis of mouse tissue samples. To detect SFTSV RNA, quantitative RT-PCR was performed according to user guidelines using the Ezplex® SFTSV Real-time PCR Kit (SML Genetree, South Korea). PCR was performed using a CFX96 instrument (Bio-Rad, Hercules, CA, USA) under the following conditions: 2 min at 25 °C, 30 min at 50 °C, and 5 min at 95 °C, followed by 40 cycles of 15 s at 95 °C and 45 s at 60 °C. Serial dilution of SFTSV DNA plasmids containing SFTSV PCR product was used to build standard curves for qPCR run. The DNA fragment was purified from the SFTSV PCR product and inserted into plasmid DNA using the pGEM-T vector system (Promega) according to the manufacturer’s protocol. Plasmid DNA was extracted using the AccuPrep® Plasmid Mini Extraction Kit (Bioneer Inc. Daejeon South Korea) and the quantity of plasmid DNA was measured on a nano spectrophotometer (Thermo Fisher Scientific). Several plasmid dilutions were tested, varying from 1 × 10^2^ to 1 × 10^10^ copies/μL. A series of log dilutions of SFTS-standard plasmid DNA from 10^6^ to 10^2^ copies/μL was then prepared, to establish an external standard curve for the qPCR. These standard control diluents were aliquoted and stored at − 70 °C or less until use.

### Focus reduction neutralization test

The 50% focus reduction neutralization test (FRNT_50_) assay was used to determine the titers of neutralizing antibodies against SFTSV in human sera. Serum samples were heat-inactivated at 56 °C for 30 min and diluted in twofold increments from 1:10 to 1:320. Each dilution was mixed with an equal volume of a solution containing SFTSV (160 FFU/0.1 mL)^11^. The mixture was inoculated into Vero E6 cells prepared in 24-well plates and incubated for 1 h at 37 °C. Culture medium was used as a control. After incubation, the cells were overlaid with 1 mL Dulbecco’s modified Eagle’s medium containing 1.5% carboxymethyl cellulose, and the cells were incubated for an additional 2 days. After 2 days of incubation, cells were fixed with 4% paraformaldehyde and incubated with 500 μL/well of anti-SFTSV NP monoclonal antibodies diluted with 0.5% Triton X-100 in PBS for 90 min at room temperature, followed by incubation with HRP-conjugated secondary antibodies. The foci of the SFTSV-infected cells were visualized using a 3,3′-Diaminobenzidine substrate kit. The plaque reduction percentage was calculated using the following formula: [(number of foci of SFTSV diluted without serum) − (number of foci of SFTSV diluted with serum)] × 100/number of foci of SFTSV diluted without serum. From this plaque reduction percentage, the FRTN_50_ titers were calculated by the [log(inhibitor) vs. normalized response] equation, using GraphPad Prism 8.0.

### NHP care and study design

Nine cynomolgus macaques (*Macaca fascicularis*; Cambodian in origin; three males and six females aged 5–7 years) from the Korea National Primate Research Centre (KNPRC) were used in this study. All animals were housed in infrastructure facilities at the Korea Research Institute of Bioscience and Biotechnology (KRIBB) under animal biosecurity level 2 (ABL-2). Macaques were anesthetized with a combination of ketamine sodium (10 mg/kg) and tiletamine/zolazepam (5 mg/kg) for immunization and blood collection. The macaques were randomly assigned to two groups (*n* = 3/group) that were balanced for sex and body weight.

Blood samples were collected in EDTA-containing tubes at 0, 2, 4, and 8 weeks post-initial vaccination. Hematological data were determined based on whole blood samples using an automatic hematology analyzer (Mindray BC-5000, Nanshan, China). All procedures were approved by the KRIBB Institutional Animal Care and Use Committee (Approval No. KRIBB-AEC-21180).

### Histological analysis

Sectioned tissues from experimental animals (G1–G4) were submerged in 10% neutral buffered formalin, dehydrated, paraffin-embedded, and sectioned at 5-μm thickness for histological examinations. The histopathological images were obtained and evaluated using Aperio ImageScope version 12.3 (Leica Biosystems Pathology Imaging, Buffalo Grove, IL, USA). The severity of histological changes was determined using a 5-point score system, as follows: 0 = no abnormality detected, 1 = minimal, 2 = mild, 3 = moderate, 4 = moderately severe, and 5 = severe. Distribution was recorded as focal, multifocal, and diffused. Recruitment of inflammatory cells in the liver and kidney and morphological alteration of the tissues (liver, kidney, and spleen) were assessed after hematoxylin and eosin staining under the light microscope.

### RNA-seq data preprocessing and analysis

Illumina paired-end reads were mapped to the primary assembly mouse genome (GRCm39) using Bowtie 2 with the local alignment option “–local”^25^. Gene quantification was performed based on the comprehensive gene annotation of mouse GENCODE release M28^26^ using featureCounts excluding chimeric fragments “-C”^27^. Genes with average read counts of < 100 were filtered out. Depth normalization and log2 transform were applied for the heatmap analysis. Differential gene expression analysis was conducted using DESeq2 version 1.24.0 with default options^28^.

CIBERSORTx^29^ and PLIER^30^ were used for estimating the immune cell composition and pathway-level information from RNA-seq data of splenic tissues from vaccinated mice. Analysis by CIBERSORTx was performed using the web server (last updated on June 15, 2022) with the built-in LM22 immune matrix file. The gene count matrix was used as input without batch correction. Quantile normalization was disabled, and 1000 permutations were used for the significance analysis. Pathway-level analysis was performed using PLIER version 0.99.0 with default parameters and the 22 immune-system pathways from the KEGG database (last updated on March 24, 2022)^31^. Data were visualized using the R package ggplot2.

### Statistical analysis

All values are expressed as mean ± standard deviation (SD). All data were statistically analyzed using the original values before being log-transformed. Statistical analyses were performed using GraphPad Prism (GraphPad Software Inc., La Jolla, CA). *P*-values were determined using one-way ANOVA with Bonferroni multiple comparison or Tukey’s multiple comparison. For the histological analysis, data are expressed as mean ± SD. Data were analyzed using a one-way ANOVA with Tukey’s post hoc test for multiple-group comparisons. The type of analysis and statistical interpretation of results are indicated in the figure legends.

## Results

### Construction and production of Ad5 expressing Tm deleted Gn gene (rAd5-GnΔTM) and stem deleted Gn protein

**Figure 1:**
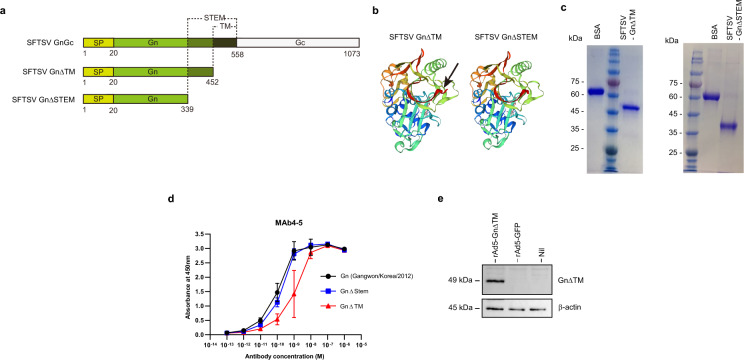
Generation and characterization of Gn protein and rAd5-Gn. (**a**) Schematic diagram of the SFTSV GnGc, Gn∆TM, and Gn∆STEM. (**b**) Predicted structure of Gn∆TM and Gn∆STEM by SWISS-MODEL^44^. A black arrow indicates a different site for GnΔTM and GnΔSTEM. (**c**) SDS-PAGE of purified GnΔTM and GnΔSTEM. (**d**) ELISA binding curves of MAb4-5 to Gn∆TM, Gn∆STEM, and Gn coated at equimolar concentrations. The average ± SD from at least two independent experiments performed is shown. (**e**) Examining the incorporation of GnΔTM into the adenovirus type 5 by western blot analysis. Un.

As the Gn protein mediates the entry of SFTSV it was decided to be used as the target protein. The transmembrane domain deleted (GnΔTM), or stem region deleted SFTSV Gn protein (GnΔSTEM) (Fig. 1a) were designed and produced to achieve a soluble protein. A Gp67 signal peptide sequence was fused with the Gn sequence to enable protein secretion from insect cells. Proteins were expressed using *Spodoptera frugiperda* (Sf9) cells and the baculovirus-mediated Bac-to-Bac expression system. Predicted 3D structures of GnΔTM and GnΔSTEM were almost identical (Fig. 1b). The molecular weight of each expressed protein, as confirmed by the SDS-PAGE assay, was approximately 49 kDa and 36 kDa (Fig. 1c), respectively. The binding affinity of GnΔTM, GnΔSTEM, and intact protein was compared using a human monoclonal antibody (MAb4-5), which shows high neutralizing activity against SFTSV Gn protein^32^. As shown in Fig. 1d, both GnΔTM and GnΔSTEM showed high binding affinity to MAb4-5. However, GnΔTM showed a lower KD value than GnΔSTEM for MAb10 (Supplement Fig. 1). Although GnΔTM showed a higher binding affinity than GnΔSTEM for MAb10, GnΔSTEM was selected as the target protein vaccine antigen because its protein production yield was 3.5-fold higher than that of GnΔTM (data not shown). From here onward, the GnΔSTEM is denoted as Gn.

Next, the recombinant adenovirus type 5 expressing transmembrane domain deleted Gn (rAd5-Gn) (Fig. 1a) was cloned and rescued in HEK293 cells. The titer of rAd5-Gn, estimated by immunohistochemical detection of the adenoviral hexon protein, was 1 × 10^11^ IU/mL. Western blot assay results showed that 49 kDa bands, corresponding to GnΔTM, were detected in rAd5-Gn-infected cells, but not in Ad5-GFP-infected cells (Fig. 1e).

### rAd5-Gn priming and Gn protein-boosting elicited humoral immunity balanced with Th1/Th2 responses

**Figure 2:**
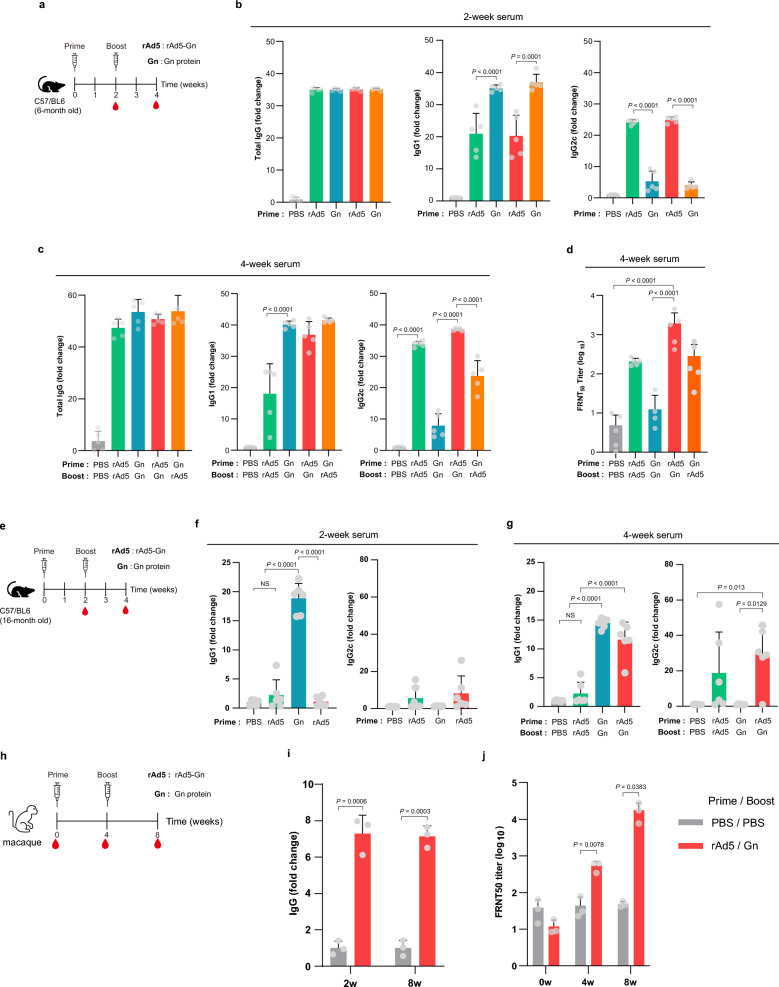
Comparison of humoral immune responses induced by homologous or heterologous vaccination with Ad5-Gn and Gn protein. (**a**) The immunization procedure and serum collection. Six-week-old C57BL/6 mice (*n* = 5/group) are immunized intramuscularly with 1 × 10^9^ infectious unit (IU) of rAd5-Gn or alum and RNA-adjuvanted Gn protein at 0 and 2 weeks. Sera are collected at indicated time points. (**b** and **c**) Total IgG, IgG1, and IgG2c are measured by indirect ELISA. (**d**) SFTSV-specific neutralizing activity of plasma (*n* = 25) from immunized mice is analyzed using the standard 50% focus reduction neutralization test (FRNT_50_). (**e**) The immunization procedure and serum collection. 16-month-old C57BL/6 mice (*n* = 5/group) are immunized intramuscularly with 1 × 10^9^ IU of rAd5-Gn or alum and RNA-adjuvanted Gn protein at 0 and 2 weeks. Sera are collected at indicated time points. (**f** and **g**) IgG1 and IgG2c were measured by indirect ELISA. (**h**) NHPs (*n* = 3/group) are intramuscularly immunized 1 × 10^9^ IU of rAd5-Gn or alum and RNA-adjuvanted Gn protein at 0 and 4 weeks. Sera are collected at indicated time points. (**i**) Total IgG was measured by indirect ELISA (**j**) SFTSV-specific neutralizing activity of plasma from immunized NHPs is analyzed using the FRNT_50_. (**b** to **c**, **f** to **g**, **i**) Data are presented as mean values ± SD. Mean fold changes relative to PBS control group. *P-*values were calculated using one-way ANOVA with Bonferroni multiple comparison test.

First, the humoral responses induced by homologous or heterologous vaccination with rAd5-Gn or Gn protein-boosting were evaluated. Mice were intramuscularly primed and boosted with rAd5-Gn (1 × 10^9^) or Gn protein-boosting (5 μg) at 2-week intervals (Fig. 2a). The Gn protein was injected with 30 μL of alum and 20 μg of RNA adjuvant. Alum was chosen as an adjuvant because of its long safety record^33^. Single-stranded RNA was used as an adjuvant to increase immunogenicity, as reported previously that it induces T-cell immune responses^15–17^. As indicated in Fig. 2b,c, all immunized groups showed similar total IgG fold change values after priming and boosting. rAd5-Gn priming induced higher levels of IgG2c (representing Th1 responses) than Gn protein priming, whereas Gn protein priming induced higher levels of IgG1 (representing Th2 responses) than rAd5-Gn priming. After boosting, the heterologous prime-boost vaccinations with rAd5-Gn and Gn protein groups showed increased IgG1 levels similar to those of the Gn protein homologous vaccination groups. Furthermore, the rAd-Gn/Gn heterologous vaccination groups showed much higher IgG2c levels than the Gn protein homologous vaccination groups (Fig. 2c). Between the heterologous vaccination groups, the rAd-Gn/Gn protein heterologous vaccination group showed higher IgG2c levels than the Gn protein/rAd-Gn vaccination groups (Fig. 2c). These results indicate that homologous immunization with the Ad5-Gn or Gn protein induced IgG2c or IgG1 biased responses, respectively, whereas heterologous prime-boosting resulted in more balanced IgG2c and IgG1 responses.

As inducing neutralizing antibodies is a requirement for successful vaccine development, the 50% focus reduction neutralization test (FRNT_50_) titers in the serum of each group were tested. Notably, rAd5-Gn/Gn protein heterologous vaccination induced the highest FRNT_50_ titers, whereas the rAd5-Gn homologous vaccination and Gn protein/rAd5-Gn heterologous vaccination groups showed similar levels of FRNT_50_ titers (FRNT_50_ titers (log10); rAd5-Gn/Gn protein: 3.1; rAd5-Gn/rAd5-Gn: 2.3; Gn protein/rAd5-Gn: 2.2). Mice that received homologous vaccination with the Gn protein showed the lowest FRNT_50_ titers (Fig. 2d).

Next, 16-month-old mice were intramuscularly primed and boosted with rAd5-Gn (1 × 10^8^ IU) or boosted with Gn protein (5 μg) at 2-week intervals (Fig. 2e). Similar to the young mice, IgG1-biased response was induced in the Gn protein priming group, whereas IgG2c response was induced in the rAd5-Gn priming group (Fig. 2f). After boosting, the rAd5-Gn/Gn protein heterologous vaccinated group showed much higher IgG1 levels than the rAd5-Gn homologous vaccinated group and higher IgG2c levels than the Gn protein homologous vaccinated group (Fig. 2g).

Non-human primates (NHPs) are used extensively to test vaccine efficacy because of their genetic similarity to humans. The efficacy of the vaccine candidates was tested in NHPs. Three NHPs intramuscularly received heterologous immunization with rAd5-Gn (1 × 10^9^ IU) and RNA adjuvant-formulated Gn (50 μg) at 4-week intervals, which we determined to be sufficient in our previous studies^15,34,35^, whereas control NHPs received phosphate-buffered saline (PBS) (Fig. 2h). As shown in Fig. 2i, Gn protein-specific IgG antibodies were detected at 2 weeks after rAd5-Gn priming and increased after Gn protein boosting. Next, it was evaluated whether heterologous immunization with rAd5-Gn (1 × 10^9^ IU) and Gn protein induced neutralizing antibodies against SFTSV. Of note, the average FRNT_50_ titers of rAd5-Gn/Gn protein heterologous vaccinated NHPs was significantly higher than those of PBS received control group (Fig. 2j).

### rAd5-Gn priming and Gn protein-boosting effectively induced the T cell responses

**Figure 3:**
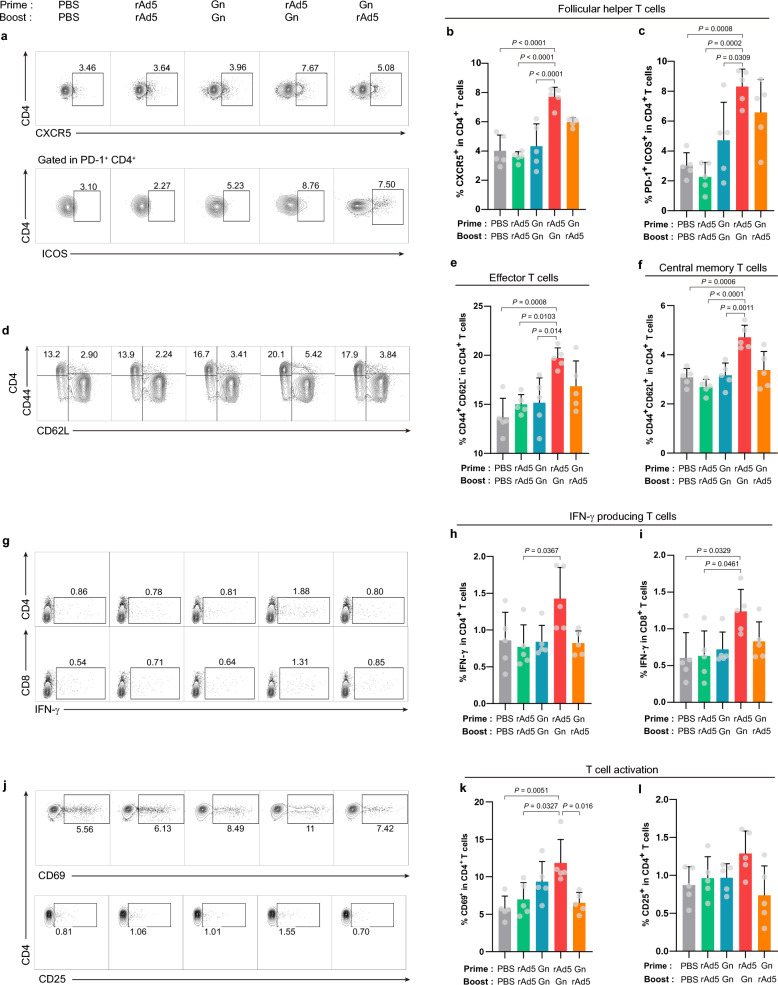
Increased T cell-mediated immune responses by rAd5-Gn priming and Gn protein boosting. Six-week-old C57BL/6 mice (*n* = 5/group) are immunized intramuscularly with 1 × 10^8^ infectious units of rAd5-Gn or alum and RNA-adjuvanted Gn protein at 0 and 2 weeks. Mice are sacrificed one week after boosting. (**a**, **d**, **g)**, and (**j**) Representative flow cytometry plots showing (**a**) follicular helper T cells, (**d**) effector T cells, central memory t cells, (**g**) interferon γ cytokine-producing T cells, (**j**) activated T cells. Frequencies of (**b**) CXCR5, (**c**) PD-1 expression in CD4^+^ T cell, and ICOS expression in CD4 + PD-1 + populations from immunized mice spleen. Frequencies of (**e**) effector memory T cells and (**f**) central memory T cells in CD4^+^ T cells were analyzed using flow cytometry. IFN-γ-producing (**h**) CD4^+^ and (**i**) CD8^+^ T cells in the spleen were analyzed using flow cytometry. (**k**) CD69 and (**l**) CD25 expression in CD4 + T cells in the spleen was analyzed using flow cytometry. Data are presented as mean values ± SD. *P* values are calculated using one-way ANOVA with Bonferroni multiple comparison test.

Follicular helper T cell (Tfh) responses reflect the development of protective high-affinity antibody responses after vaccination. Thus, it was checked whether enhanced neutralizing antibody responses induced by rAd5-Gn priming and Gn protein-boosting were associated with the increase in or activation of Tfh. Mice were intramuscularly primed and boosted with rAd5-Gn (1 × 10^8^ IU) or boosted with the Gn protein (5 μg) at 2-week intervals and were sacrificed one week after boosting (Supplementary Fig. 1). As depicted in Fig. 3a,b, increased frequencies of CD4^+^CXCR5^+^ Tfh were observed in both heterologous prime-boosted groups (rAd5-Gn/Gn protein and Gn protein/rAd5-Gn). Further, the frequency of CD4^+^PD-1^+^ICOS^+^ Tfh in immunized mice was checked because ICOS has previously been used to monitor Tfh responses in several different studies^36–38^. Interestingly, the percentages of ICOS^+^ Tfh were strongly increased in the rAd5-Gn/Gn protein group (Fig. 3a,c). In CD4^+^ T cells, the percentage of effector memory T cell (Tem) significantly increased only in the rAd5-Gn/Gn protein heterologous vaccination group (Fig. 3d,e). There was no significant increase in the central memory T cell (Tcm) of CD8^+^ T cells in any of the groups (data not shown). Importantly, CD4^+^Tcm populations were only dramatically increased in the rAd5-Gn/Gn protein heterologous vaccination group (Fig. 3d,f). Similar to these results, proliferating (Ki-67^+^) Tem and Tcm increased only in the rAd5-Gn/Gn protein heterologous vaccinated group (Supplementary Fig. 2).

Antigen-specific interferon (IFN)-γ producing CD4^+^ and CD8^+^ T cells were increased in the rAd5-Gn/Gn protein heterologous vaccinated group (Fig. 3g–i). In addition, rAd5-Gn/Gn protein heterologous vaccination induced more balanced Th1/Th2 cytokine responses. The highest levels of IFN-γ, IL-12p70, IL-15, IL-22, IL-2, IL-4, IL-10, IL-6, and TNF-α/β production were detected in splenocyte culture supernatants of the rAd5-Gn/Gn protein heterologous vaccinated group (Supplementary Fig. 3).

Furthermore, CD25 and CD69 expression on CD4^+^ T cells was increased in the heterologous rAd5-Gn/Gn protein group (Fig. 3j–l). CD8^+^ T cells from the homologous rAd5-Gn/rAd5-Gn group expressed high levels of killer cell lectin-like receptor G1 (KLRG1), which is known to be a marker of antigen-experienced cells or short-lived effector cells (Supplementary Fig. 2).

In 16-month-old mice, although the activity of interferon-γ-producing cells was highest in the homologous rAd5-Gn immunized group, the rAd5-Gn/Gn protein heterologous vaccinated group also showed significantly increased activity of interferon-γ-producing cells compared to the Gn protein homologous vaccinated group (Supplementary Fig. 4).

### rAd5-Gn priming and Gn boosting provided complete protection against the lethal SFTSV challenge in mice

**Figure 4:**
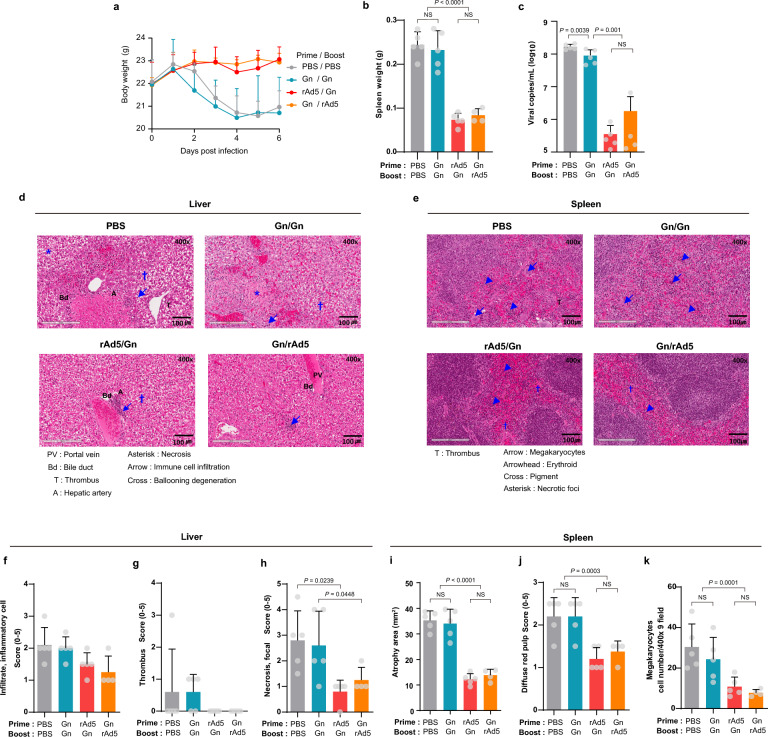
Protective efficacy of Ad5-Gn priming and Gn protein boosting. Six-week-old C57/BL6 wildtype mice (PBS/PBS (*n* = 5), Gn/Gn (*n* = 5), rAd5-Gn (*n* = 5), Gn/rAd5 (*n* = 4)) immunized with rAd5-Gn (1 × 10^8^ infectious unit) and/or Gn protein intramuscularly at 2-week intervals and challenged with SFTS virus at 5 weeks after the second immunization (Fig. S1c). The mice received the anti-interferon receptor antibody and IL-10 at 4 days and 1 day before the virus challenge. (**a**) Weight loss of mice. (**b**) Weights of the spleen of mice. (**c**) Quantitative real-time PCR was used to measure viral genomic RNA from the spleen of challenged mice. (**d** to **k**) Pathological changes in the liver and spleen of mice infected with SFTSV. Representative Hematoxylin and Eosin-stained tissue sections of the (**d**) liver and (**e**) spleen from SFTSV-infected mice. (**f**) The scores for inflammatory cell infiltration, (**g**) thrombus, and (**h**) necrosis in the liver. (**i**) Area of the cross-section of the spleen and (**k**) number of megakaryocytes evaluated using histological images obtained from a slide scanner. (**j**) Diffuse white pulp in the spleen was determined using a 5-point score system, as follows: 0 = no abnormality detected (NAD), 1 = minimal, 2 = mild, 3 = moderate, 4 = moderately severe, and 5 = severe. *P*-values are calculated using one-way ANOVA with Bonferroni multiple comparison and Tukey’s multiple comparison or Mann–Whitney U test.

Next, the protective efficacy of the vaccine candidates was investigated. Because normal wild-type mice are not susceptible to SFTSV, the mice in this study received the anti-interferon receptor antibody and IL-10 before the virus challenge to temporally suppress their immune response^39^ (Supplementary Fig. 1). After the challenge, the rAd5-Gn/Gn protein heterologous vaccinated group showed an increase in body weight, whereas the control PBS group and Gn protein homologous vaccinated group showed significant weight loss (Fig. 4a). Mice were sacrificed six days after the challenge, and spleen weight and viral copies in the spleen were measured. The spleen weight of the rAd5-Gn/Gn protein heterologous vaccinated groups was significantly lower than that of the control PBS group and Gn protein homologous vaccinated group (Fig. 4b). Furthermore, viral copy numbers in the spleen were the lowest in the rAd5-Gn/Gn protein heterologous vaccinated group, followed by the Gn protein/rAd5-Gn heterologous vaccinated group (Fig. 4c).

In line with these results, in the liver (Fig. 4d), inflammatory cell infiltration and thrombus were slightly decreased in the rAd5-Gn and Gn protein heterologous vaccinated groups (Fig. 4f,g), and hepatocyte necrosis with shrinking nucleic or hepatocyte degradation was significantly decreased in the rAd5-Gn/Gn protein heterologous vaccinated group (Fig. 4h). During SFTSV infection, pathological changes were mainly identified in the spleen (Fig. 4e). The atrophy area was significantly reduced in both the rAd5-Gn/Gn protein and the Gn protein/rAd5-Gn heterologous vaccinated groups (Fig. 4i). Contrastingly, no significant reduction of atrophy in the Gn/Gn protein homologous vaccinated group was found. There was a visible increase in diffuse red pulp in infected spleens, which is indicative of reticuloendothelial hyperplasia of red pulp (Fig. 4j). The reticuloendothelial system plays an important role in spleen defense by removing viruses. The diffuse red pulp means there are still more viruses in the PBS and homologous vaccinated groups as compared with heterologous vaccinated groups. Increase in mononuclear cells and depletion in the red pulp might coincide with the increase in megakaryocytes observed in the spleen, a secondary hematopoietic organ in mice, based on their cellular morphology (Fig. 4k). Thus, these findings in the spleen indicated that a heterologous vaccination strategy is an effective way to protect tissues against SFTSV via the promotion of rapid viral clearance.

### Heterologous prime-boost regimen enables the timely activation of distinct immune pathways

**Figure 5:**
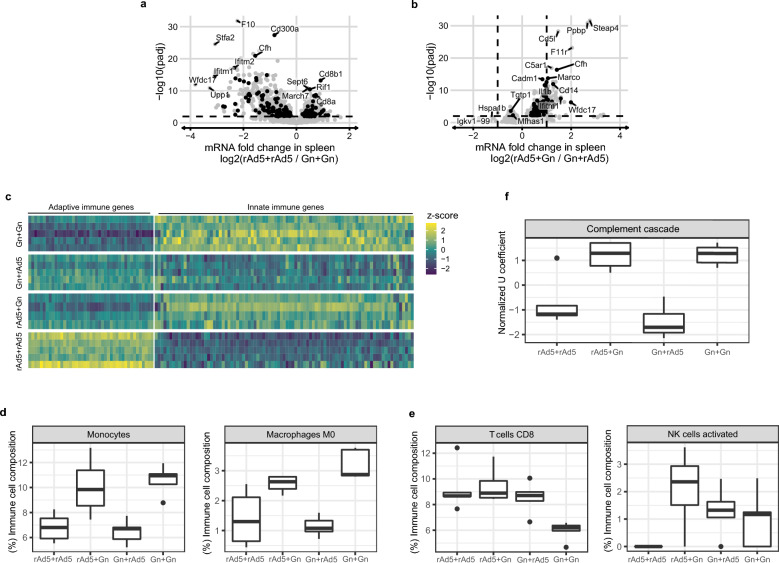
RNA-seq analysis reveals that rAd5 and Gn each regulate distinct biological pathways. Differential expression analysis (**a**) between rAd5 + rAd5 (*n* = 5) and Gn + Gn (*n* = 5) and (**b**) between rAd5 + Gn (*n* = 4) and Gn + rAd5 (*n* = 5) vaccinated mice. The horizontal dashed line indicates the significant threshold (adjusted *P*-value < 0.01). Differentially expressed genes related to adaptive or innate immune response are marked in black. (**c**) Normalized expression values of adaptive and innate immune genes. Z-scores were computed for each gene for data visualization. (**d** and **e**) Immune cell composition estimation by CIBERSORTx and (**f**) immune KEGG pathway-level analysis by PLIER from RNA-seq data of splenic tissues of homologous and heterologous vaccinated mice.

To investigate the underlying mechanism of the heterologous prime-boost regimen, RNA-seq of the splenic tissues of mice undergoing different vaccination regimens was conducted 3 days after boosting. Differential analysis revealed that adaptive immune genes, such as *CD8a*, *Cd8b1*, *March7*, and *Rif,* were increased in the rAd5-Gn/rAd5-Gn homologous vaccinated group as compared with those in the Gn/Gn protein homologous vaccinated group (Fig. 5a). Conversely, innate immune genes, such as *Cd300a*, *Cfh*, *Ifitm1*, *Ifitm2*, and *Wfdc17,* were upregulated in the Gn/Gn vaccinated group. Interestingly, CD14, pro-platelet basic protein (Ppbp), and Macro genes related to monocyte/macrophages were highly increased in the rAd5-Gn/Gn protein heterologous vaccinated group as compared with those in the Gn/rAd5-Gn heterologous vaccinated group (Fig. 5b). Notably, the cell adhesion molecule Cadm1 regulates susceptibility to the cytotoxicity mediated by natural killer cells^40^.

Heatmap analysis revealed that immunization with Gn protein increased the innate immune response-related genes, whereas rAd5-Gn immunization increased the adaptive immune response-related genes (Fig. 5c). Homologous Gn protein immunization induced the highest expression of innate immune response-related genes, whereases homologous rAd5-Gn immunization induced the highest expression of adaptive immune response-related genes. In line with the above results, monocyte/macrophage and complement cascade genes related to innate immune response were increased in the Gn protein-boosted groups (Fig. 5d,f). While the Gn/Gn protein homologous vaccinated group showed lower expression levels of CD8^+^ T cell response-related genes, the rAd5-Gn/Gn protein heterologous vaccinated group showed similar levels of CD8^+^ T cell response-related gene expressions, as compared with the rAd5-Gn/rAd5-Gn or Gn/rAd5-Gn group (Fig. 5e). Interestingly, activated NK cell-related genes were highly expressed in the rAd5-Gn/Gn protein heterologous vaccinated group.

## Discussion

Despite its importance, a clinically approved effective vaccine for SFTS has not yet been developed. Heterologous prime-boost immunization is a vaccination protocol comprising different delivery systems that express the same or overlapping antigenic inserts. Although emerging evidence, such as a comparison of the effects of different types of SARS-CoV-2 vaccine, has shown that heterologous vaccine strategy generates considerably higher immune responses^41^; a deeper mechanism of action has not been clearly defined. This study evaluated the vaccine potential of heterologous rAd5-Gn prime followed by Gn protein boost and examined the immunological mechanism underlying the improved vaccine response to this strategy for SFTS. In contrast to homologous vaccination, heterologous vaccination induced a balanced IgG1 (indicated Th2)/IgG2c (indicated Th1) antibody response in mice.

Remarkably, rAd5-Gn/Gn protein heterologous immunization elicited higher levels of FRNT_50_ titers, which significantly exceeded those of both recovered patients and those with homologous immunization in mice. Interestingly, the reverse heterologous prime-boost regimen (Gn protein prime/rAd5-Gn boost) did not reach a similar level of neutralizing antibody and IgG2c in the rAd5-Gn/Gn protein group in mice. In line with these results, activated follicular helper T cells were also increased only in the rAd5-Gn/Gn protein heterologous immunized group. Thus, the sequence of administration of vectors is also a critical factor in determining the extent of immune responses, similar to the results observed in other studies^23^.

Of note, rAd5-Gn/Gn protein heterologous immunization elicited a balanced IgG1/Ig2c humoral response even in 16-month-old mice. Therefore, these results indicate that rAd5-Gn priming and the Gn protein-boosting strategy is an effective way to achieve humoral immunity with optimal Th1/Th2 balance in both young and old mice. Impaired vaccine-induced immune responses in the elderly are associated with immunosenescence^42^. This result indicated that the heterologous prime-boost strategy may elicit proper immune responses in the elderly by overcoming immunosenescence. As most fatal cases of SFTS occur in the elderly, these results imply that the heterologous prime-boost strategy could be an effective vaccine candidate against SFTS for these patients.

In addition to humoral responses, rAd5-Gn/Gn protein heterologous immunization induced strong T-cell responses. The frequency of effector/central memory T cells was significantly increased in rAd5-Gn/Gn protein heterologous immunization. In line with this result, IFN-γ producing T cells, as well as activated T cells were highest in rAd5-Gn/Gn protein heterologous immunized group. As T cells play a crucial role in virus elimination and protection from viral infections, it is believed that rAd5-Gn/Gn protein heterologous vaccination-induced T cell responses could provide effective protection against viral infection. Furthermore, it is expected to protect against mutated SFTSV variants because T cells recognize more conserved nonmutable viral epitopes than antibodies. Although a challenge experiment was not conducted in aged mice, the FRNT50 levels of aged mice were between 256 and 512. Considerably increased levels of FRNT50 were observed in rAd5-Gn-immunized mice compared to those of control mice, and these values were similar to those of SFTSV survivors even though these are relative values. Furthermore, the numbers of IFN-γ-producing cells were also significantly increased in aged mice immunized with rAd5-Gn/Gn. Thus, we believe that increasing the cellular and humoral responses in aged mice by rAd5-Gn/Gn could provide protection against SFTSV infection.

Based on RNA-seq analysis, it appears that rAd5-Gn immunization predominantly activates genes related to adaptive immune responses, whereas Gn protein immunization activates genes related to innate immune responses.

In this study, the Gn protein was formulated with alum and RNA adjuvant to maximize efficacy. Although we could not assert whether the increased innate immune response pathway by Gn protein immunization was dependent on RNA adjuvant, this is still possible since previous studies have demonstrated that RNA adjuvant can stimulate the innate immune response pathway^17^. In addition, alum was revealed to induce NETosis and the DAMP pathway to exert its adjuvant effects^43^. Thus, we believe that the prominent increase in the innate immune response induced by the Gn protein might be due to the alum and RNA adjuvant. We found several immune response-related genes that specifically increased according to the type of priming, such as *CD8a, CD8a, Cd8b1, March7*, and *Rif*. Additionally, we found that several genes, including *Hspa1b, Igkv1-99, Sept6, UPP1, Stfa2*, *F10, Steap4, Cd5l, F11r,* and *C5ar1,* were differentially regulated in different vaccinations, but their immunological roles remain unclear because their functions were not directly associated with immune cells. However, they might affect the immune response indirectly, and further studies might identify their effects.

It has been shown earlier that the activation of innate immune response contributes to increased vaccine efficacy^15^. Consequently, the heterologous rAd5-Gn/Gn protein prime-boost strategy induced both humoral and cell-mediated immune responses via relatively unbiased activation of adaptive and innate immune pathways, compared to homologous immunization, which is essential for the development of effective vaccines. Notably, reverse heterologous prime-boost (Gn protein/rAd5-Gn) did not induce strong innate and adaptive immune response-related gene expressions. While the rAd5-Gn/Gn prime-boost strategy could induce potent CD8^+^ and NK cells as well as macrophage/monocyte-related gene expression, reverse immunization failed to reach the monocyte/macrophage gene expression levels of the rAd5-Gn/Gn protein group.

In the present study, we first designed rAd5 and protein subunit type SFTSV vaccines and tested their efficacy for homologous and heterologous immunization. Despite its clinical importance, only a few SFTSV vaccine candidates have been developed and tested. In addition, none of the previous studies tested the protein subunit type SFTSV vaccine. Furthermore, we demonstrated that although the homologous immunization of the Gn subunit vaccine failed to induce potent immune responses and did not protect against SFTSV challenge, heterologous rAd5-Gn prime and Gn protein-boosting elicited more balanced adaptive and innate immune responses and provided protection. Nevertheless, further studies are needed to clarify the efficacy of rAd5-Gn/Gn immunization in aged mice and NHP models. Although we confirmed that rAd-Gn/Gn immunization induced high levels of neutralizing antibodies in aged mice and NHPs, additional virus-challenge experiments would be helpful to confirm the protective efficacy of heterologous prime-boost in aged mice and NHP models. Furthermore, to optimize the dose of rAd5-Gn, the protective efficacy of various doses should be tested.

In addition, for the clinical application of rAd5-Gn/Gn heterologous prime-boost, several aspects should be considered. Even though several studies have shown that heterologous prime-boost can induce strong and more durable immune responses than homologous immunization, manufacturing different types of vaccines can lead to increased burden in terms of manufacturing, storage, and distribution cost, as well as management. Moreover, our study demonstrated that homologous immunization with rAd5-Gn can induce considerable levels of FRNT_50_, although they were lower than those of the rAd5-Gn/Gn protein in the mouse model. Therefore, homologous immunization with rAd5-Gn may be more advantageous than rAd5-Gn/Gn protein in terms of economics and convenience. However, homologous rAd5-Gn immunization may not be suitable for individuals who already have high levels of antibodies against the rAd5 vaccine. Few studies using viral vector vaccines have reported that pre-existing immunity can hamper vaccine-induced immune responses. Pre-existing immunity against human adenovirus serotype 5 is widespread in the human population. However, to prove this, it is essential to confirm whether rAd5-Gn/Gn can induce sufficient protective immunity in mice with pre-existing immunity to rAd5-Gn. Additionally, it is necessary to elucidate more precise immunization intervals and dosages under the above conditions. Moreover, it is generally accepted that subunit vaccines are one of the safest vaccines. Therefore, rAd5-Gn/Gn immunity can be an alternative for individuals who already have antibodies to rAd5 or have experienced side effects. Since rAd5-Gn/Gn induces potent immune responses compared to homologous immunization with rAd5-Gn, it may be more suitable for immunocompromised individuals who are unable to achieve sufficient protective immune responses.

In conclusion, the present study findings showed that a proper heterologous prime-boost (viral vector vaccine as prime and protein vaccine as boost) regimen is an effective SFTS vaccine development strategy, as it induced balanced Th1/Th2 responses and high titer of neutralizing antibodies, as well as robust T cell responses.

## Supplementary Information

Supplementary Information.

## Data Availability

The authors declare that the data supporting the findings of this study are available within the paper and its supplementary information files. Source data are provided with this paper.
