# Supplementary material for: Heterologous vaccination utilizing viral vector and protein platforms confers complete protection against SFTSV
Source: Sci Rep. 2023 May 20;13:8189. doi: 10.1038/s41598-023-35328-9 (PMC10199661; doi:10.1038/s41598-023-35328-9)
Supplement: Supplementary file 1 — Supplementary Information. [file 41598_2023_35328_MOESM1_ESM.docx]

***Supplementary Information for***

**Heterologous vaccination utilizing viral vector and protein platforms confers complete protection against SFTSV**

Jae-Yong Kim^*^, Kyeongseok Jeon^*^, Jung Joo Hong^*^, Sang-In Park^*^, Hyeonggon Cho^*^, Hyo-Jung Park^,*^, Hye Won Kwak, Hyeong-Jun Park, Yoo-Jin Bang, Yu-Sun Lee, Seo-Hyeon Bae, So-Hee Kim, Kyung-Ah Hwang, Dae-Im Jung, Seong Hoo Cho, Sang Hwan Seo, Green Kim, Hanseul Oh, Hwal-Yong Lee, Ki Hyun Kim, Hee-Young Lim, Pyeonghwa Jeon, Joo-Yeon Lee, Junho Chung, Sang-Myeong Lee, Hae Li Ko, Manki Song, Nam-Hyuk Cho^*^, Young-suk Lee^*^, So-Hee Hong^*^, Jae-Hwan Nam^*^

**Supplementary Materials and Methods**

**Sequence of baculovirus expression vector**

The full sequence of the baculovirus vector expressing SFTSV Gn is presented below, with the SFTSV Gn sequence underlined.

GACGCGCCCTGTAGCGGCGCATTAAGCGCGGCGGGTGTGGTGGTTACGCGCAGCGTGACCGCTACACTTGCCAGCGCCCTAGCGCCCGCTCCTTTCGCTTTCTTCCCTTCCTTTCTCGCCACGTTCGCCGGCTTTCCCCGTCAAGCTCTAAATCGGGGGCTCCCTTTAGGGTTCCGATTTAGTGCTTTACGGCACCTCGACCCCAAAAAACTTGATTAGGGTGATGGTTCACGTAGTGGGCCATCGCCCTGATAGACGGTTTTTCGCCCTTTGACGTTGGAGTCCACGTTCTTTAATAGTGGACTCTTGTTCCAAACTGGAACAACACTCAACCCTATCTCGGTCTATTCTTTTGATTTATAAGGGATTTTGCCGATTTCGGCCTATTGGTTAAAAAATGAGCTGATTTAACAAAAATTTAACGCGAATTTTAACAAAATATTAACGTTTACAATTTCAGGTGGCACTTTTCGGGGAAATGTGCGCGGAACCCCTATTTGTTTATTTTTCTAAATACATTCAAATATGTATCCGCTCATGAGACAATAACCCTGATAAATGCTTCAATAATATTGAAAAAGGAAGAGTATGAGTATTCAACATTTCCGTGTCGCCCTTATTCCCTTTTTTGCGGCATTTTGCCTTCCTGTTTTTGCTCACCCAGAAACGCTGGTGAAAGTAAAAGATGCTGAAGATCAGTTGGGTGCACGAGTGGGTTACATCGAACTGGATCTCAACAGCGGTAAGATCCTTGAGAGTTTTCGCCCCGAAGAACGTTTTCCAATGATGAGCACTTTTAAAGTTCTGCTATGTGGCGCGGTATTATCCCGTATTGACGCCGGGCAAGAGCAACTCGGTCGCCGCATACACTATTCTCAGAATGACTTGGTTGAGTACTCACCAGTCACAGAAAAGCATCTTACGGATGGCATGACAGTAAGAGAATTATGCAGTGCTGCCATAACCATGAGTGATAACACTGCGGCCAACTTACTTCTGACAACGATCGGAGGACCGAAGGAGCTAACCGCTTTTTTGCACAACATGGGGGATCATGTAACTCGCCTTGATCGTTGGGAACCGGAGCTGAATGAAGCCATACCAAACGACGAGCGTGACACCACGATGCCTGTAGCAATGGCAACAACGTTGCGCAAACTATTAACTGGCGAACTACTTACTCTAGCTTCCCGGCAACAATTAATAGACTGGATGGAGGCGGATAAAGTTGCAGGACCACTTCTGCGCTCGGCCCTTCCGGCTGGCTGGTTTATTGCTGATAAATCTGGAGCCGGTGAGCGTGGGTCTCGCGGTATCATTGCAGCACTGGGGCCAGATGGTAAGCCCTCCCGTATCGTAGTTATCTACACGACGGGGAGTCAGGCAACTATGGATGAACGAAATAGACAGATCGCTGAGATAGGTGCCTCACTGATTAAGCATTGGTAACTGTCAGACCAAGTTTACTCATATATACTTTAGATTGATTTAAAACTTCATTTTTAATTTAAAAGGATCTAGGTGAAGATCCTTTTTGATAATCTCATGACCAAAATCCCTTAACGTGAGTTTTCGTTCCACTGAGCGTCAGACCCCGTAGAAAAGATCAAAGGATCTTCTTGAGATCCTTTTTTTCTGCGCGTAATCTGCTGCTTGCAAACAAAAAAACCACCGCTACCAGCGGTGGTTTGTTTGCCGGATCAAGAGCTACCAACTCTTTTTCCGAAGGTAACTGGCTTCAGCAGAGCGCAGATACCAAATACTGTCCTTCTAGTGTAGCCGTAGTTAGGCCACCACTTCAAGAACTCTGTAGCACCGCCTACATACCTCGCTCTGCTAATCCTGTTACCAGTGGCTGCTGCCAGTGGCGATAAGTCGTGTCTTACCGGGTTGGACTCAAGACGATAGTTACCGGATAAGGCGCAGCGGTCGGGCTGAACGGGGGGTTCGTGCACACAGCCCAGCTTGGAGCGAACGACCTACACCGAACTGAGATACCTACAGCGTGAGCATTGAGAAAGCGCCACGCTTCCCGAAGGGAGAAAGGCGGACAGGTATCCGGTAAGCGGCAGGGTCGGAACAGGAGAGCGCACGAGGGAGCTTCCAGGGGGAAACGCCTGGTATCTTTATAGTCCTGTCGGGTTTCGCCACCTCTGACTTGAGCGTCGATTTTTGTGATGCTCGTCAGGGGGGCGGAGCCTATGGAAAAACGCCAGCAACGCGGCCTTTTTACGGTTCCTGGCCTTTTGCTGGCCTTTTGCTCACATGTTCTTTCCTGCGTTATCCCCTGATTCTGTGGATAACCGTATTACCGCCTTTGAGTGAGCTGATACCGCTCGCCGCAGCCGAACGACCGAGCGCAGCGAGTCAGTGAGCGAGGAAGCGGAAGAGCGCCTGATGCGGTATTTTCTCCTTACGCATCTGTGCGGTATTTCACACCGCAGACCAGCCGCGTAACCTGGCAAAATCGGTTACGGTTGAGTAATAAATGGATGCCCTGCGTAAGCGGGTGTGGGCGGACAATAAAGTCTTAAACTGAACAAAATAGATCTAAACTATGACAATAAAGTCTTAAACTAGACAGAATAGTTGTAAACTGAAATCAGTCCAGTTATGCTGTGAAAAAGCATACTGGACTTTTGTTATGGCTAAAGCAAACTCTTCATTTTCTGAAGTGCAAATTGCCCGTCGTATTAAAGAGGGGCGTGGCCAAGGGCATGGTAAAGACTATATTCGCGGCGTTGTGACAATTTACCGAACAACTCCGCGGCCGGGAAGCCGATCTCGGCTTGAACGAATTGTTAGGTGGCGGTACTTGGGTCGATATCAAAGTGCATCACTTCTTCCCGTATGCCCAACTTTGTATAGAGAGCCACTGCGGGATCGTCACCGTAATCTGCTTGCACGTAGATCACATAAGCACCAAGCGCGTTGGCCTCATGCTTGAGGAGATTGATGAGCGCGGTGGCAATGCCCTGCCTCCGGTGCTCGCCGGAGACTGCGAGATCATAGATATAGATCTCACTACGCGGCTGCTCAAACCTGGGCAGAACGTAAGCCGCGAGAGCGCCAACAACCGCTTCTTGGTCGAAGGCAGCAAGCGCGATGAATGTCTTACTACGGAGCAAGTTCCCGAGGTAATCGGAGTCCGGCTGATGTTGGGAGTAGGTGGCTACGTCTCCGAACTCACGACCGAAAAGATCAAGAGCAGCCCGCATGGATTTGACTTGGTCAGGGCCGAGCCTACATGTGCGAATGATGCCCATACTTGAGCCACCTAACTTTGTTTTAGGGCGACTGCCCTGCTGCGTAACATCGTTGCTGCTGCGTAACATCGTTGCTGCTCCATAACATCAAACATCGACCCACGGCGTAACGCGCTTGCTGCTTGGATGCCCGAGGCATAGACTGTACAAAAAAACAGTCATAACAAGCCATGAAAACCGCCACTGCGCCGTTACCACCGCTGCGTTCGGTCAAGGTTCTGGACCAGTTGCGTGAGCGCATACGCTACTTGCATTACAGTTTACGAACCGAACAGGCTTATGTCAACTGGGTTCGTGCCTTCATCCGTTTCCACGGTGTGCGTCACCCGGCAACCTTGGGCAGCAGCGAAGTCGAGGCATTTCTGTCCTGGCTGGCGAACGAGCGCAAGGTTTCGGTCTCCACGCATCGTCAGGCATTGGCGGCCTTGCTGTTCTTCTACGGCAAGGTGCTGTGCACGGATCTGCCCTGGCTTCAGGAGATCGGAAGACCTCGGCCGTCGCGGCGCTTGCCGGTGGTGCTGACCCCGGATGAAGTGGTTCGCATCCTCGGTTTTCTGGAAGGCGAGCATCGTTTGTTCGCCCAGGACTCTAGCTATAGTTCTAGTGGTTGGCTACGTATACTCCGGAATATTAATAGATCATGGAGATAATTAAAATGATAACCATCTCGCAAATAAATAAGTATTTTACTGTTTTCGTAACAGTTTTGTAATAAAAAAACCTATAAATATTCCGGATTATTCATACCGTCCCACCATCGGGCGCGGATCC***ATGGTCCTGGTGAACCAGTCCCACCAGGGTTTCAACAAGGAGCACACCTCTAAGATGGTGTCCGCTATCGTCCTGTACGTGCTGCTCGCTGCCGCTGCCCACTCTGCTTTCGCTGACACTGGACCAATCATCTGCGCTGGACCAATCCACTCCAACAAGAGCGCCAACATCCCACACCTGCTGGGCTACTCTGAGAAGATTTGCCAGATCGACCGCCTGATCCACGTGTCCAGCTGGCTGCGTAACCACTCACAGTTCCAAGGTTACGTGGGACAGCGCGGTGGTCGCTCCCAGGTGTCCTACTACCCTGCTGAAAACTCATACTCCCGTTGGTCTGGACTGCTCAGCCCATGCGACGCTGACTGGCTGGGAATGCTGGTGGTCAAGAAGGCCAAGGGTTCCGACATGATCGTCCCAGGTCCTAGCTACAAGGGCAAGGTGTTCTTCGAAAGGCCTACCTTCGACGGATACGTGGGTTGGGGATGCGGTTCCGGCAAGAGCAGAACCGAGTCTGGAGAACTGTGCTCTTCAGACTCAGGAACTTCCAGCGGTCTGCTGCCAAGCGACAGGGTCCTGTGGATCGGCGACGTGGCTTGCCAGCCTATGACCCCCATCCCAGAGGAAACTTTCCTGGAGCTGAAGAGCTTCTCTCAGTCAGAGTTCCCTGACATCTGCAAGATCGACGGAATCGTGTTCAACCAGTGCGAGTCCGAAAGCCTGCCTCAACCCCTGGACGTGGCTTGGATGGACGTGGGTCACTCCCACAAGATCATCATGAGAGAGCACAAGACCAAGTGGGTCCAGGAATCTTCATCCAAGGACTTCGTGTGCTACAAGGAGGGCACTGGTCCCTGCTCTGAATCAGAGGAAAGGACCTGCAAGACTTCCGGCAGCTGCAGAGGAGACATGCAGTTCTGCAAGGTGGCTGGTTGCGAGCACGGTGAAGAGGCTTCCGAAGCCAAGTGCCGCTGCTCACTGGTCCACAAGCCCGGTGAAGTGGTCGTGTCCTACGGAGGTATGCGCGTGCGTCCAAAGTGCTACGGCTTCAGCCGCATGATGGCCACCCTGGAGGTGCATCACCATCATCACCATTAG***CTCGAGGCATGCGGTACCAAGCTTGTCGAGAAGTACTAGAGGATCATAATCAGCCATACCACATTTGTAGAGGTTTTACTTGCTTTAAAAAACCTCCCACACCTCCCCCTGAACCTGAAACATAAAATGAATGCAATTGTTGTTGTTAACTTGTTTATTGCAGCTTATAATGGTTACAAATAAAGCAATAGCATCACAAATTTCACAAATAAAGCATTTTTTTCACTGCATTCTAGTTGTGGTTTGTCCAAACTCATCAATGTATCTTATCATGTCTGGATCTGATCACTGCTTGAGCCTAGGAGATCCGAACCAGATAAGTGAAATCTAGTTCCAAACTATTTTGTCATTTTTAATTTTCGTATTAGCTTACGACGCTACACCCAGTTCCCATCTATTTTGTCACTCTTCCCTAAATAATCCTTAAAAACTCCATTTCCACCCCTCCCAGTTCCCAACTATTTTGTCCGCCCACAGCGGGGCATTTTTCTTCCTGTTATGTTTTTAATCAAACATCCTGCCAACTCCATGTGACAAACCGTCATCTTCGGCTACTTTTTCTCTGTCACAGAATGAAAATTTTTCTGTCATCTCTTCGTTATTAATGTTTGTAATTGACTGAATATCAACGCTTATTTGCAGCCTGAATGGCGAATGG

**DNA sequences of Ad5-Gn (GOI sequence)**

The SFTSV Gn sequence in the adenoviral vector is presented below, with the SFTSV Gn sequence underlined.

GCGCCGCCACC***ATGATGAAAGTGATTTGGTTCTCCTCTCTGATTTGTCTGGTCATTCAGTGTAGCGGGGATTCTGGACCTATTATCTGTGCTGGGCCAATCCACAGCAACAAGAGCGCCTCCATCCCCCACCTGCTGGGCTACTCCGAGAAGATCTGCCAGATCGACCGCCTGATCCACGTGAGCTCCTGGCTGCGGAACCACAGCCAGTTCCAGGGATACGTGGGACAGAGGGGAGGCCGCAGCCAGGTGTCCTACTATCCAGCCGAGAATTCTTATAGCAGATGGTCCGGCCTGCTGTCTCCATGTGACGCAGATTGGCTGGGCATGCTGGTGGTGAAGAAGGCCAAGGGCTCTGATATGATCGTGCCTGGCCCAAGCTACAAGGGCAAGGTGTTCTTTGAGCGGCCCACCTTCGACGGATATGTGGGATGGGGATGCGGATCTGGCAAGAGCAGGACAGAGTCCGGCGAGCTGTGCAGCAGCGATTCTGGCACCTCCTCTGGCCTGCTGCCTAGCGATCGCGTGCTGTGGATCGGCGACGTGGCATGCCAGCCAATGACACCCATCCCTGAGGAGACATTCCTGGAGCTGAAGTCCTTCTCTCAGAGCGAGTTTCCTGATATCTGCAAGATCGACGGCATCGTGTTCAATCAGTGTGAGGGCGAGAGCCTGCCACAGCCCTTTGATGTGGCCTGGATGGACGTGGGCCACTCCCACAAGATCATCATGCGGGAGCACAAGACCAAGTGGGTGCAGGAGAGCTCCTCTAAGGACTTCGTGTGCTACAAGGAGGGCACAGGCCCATGTTCCGAGTCTGAGGAGAAGGCCTGCAAGACCAGCGGCTCCTGTAGAGGCGATATGCAGTTTTGCAAGGTGGCAGGATGTGAGCACGGAGAGGAGGCCTCTGACGCCAAGTGCAGGTGTAGCCTGGTGCACAAGCCAGGAGAGGTGGTGGTGTCTTACGGAGGAATGCGGGTGCGGCCCAAGTGCTATGGCTTCAGCAGAATGATGGCCACACTGGAGGTGAACCCCCCTGAGCAGAGGATCGGCCAGTGCACCGGCTGTCACCTGGAGTGTATCAATGGCGGCGTGAGGCTGATCACCCTGACAAGCGAGCTGAAGTCCGCCACAGTGTGCGCCAGCCACTTCTGTAGCTCCGCCACATCTGGCAAGAAGAGCACCGAGATCCAGTTTCACTCTGGCAGCCTGGTGGGCAAGACCGCAATCCACGTGAAGGGCGCCCTGGTGGATGGCACAGAGTTCACCTTTGAGGGCTCCTGCATGTTCCCAGACGGCTGTGATGCCGTGGACTGCACCTTCTGTAGAGAGTTTCTGAAGAACCCACAGTGCTACCCCGCCAAGAAGTGA***TGTTTAAAC

**Preparation of infectious recombinant baculovirus**

Recombinant virus and protein were produced in an insect cell system. The recombinant transfer vectors were transformed to 7.5 × 10^5^ cells/ml SF9 cells (BD Bioscience) using the calcium phosphate method, and baculovirus was simultaneously infected into the insect cell. Over two consecutive subcultures, we obtained high-titer viruses in the supernatants. For recombinant protein expression, new SF9 cells (2 × 10^6^ cells/ml, 200 ml) were infected with the supernatants containing recombinant virus and cultured for 3 days (27°C, 115 rpm). Then, the culture media were collected and concentrated using the ammonium sulfate precipitation method (7 M ammonium sulfate). The precipitate was re-dissolved in a buffer solution (20 mM Tris-HCl (pH 8.0), 0.5 M NaCl, 5 mM imidazole), and then we purified histidine-tagged proteins using a nickel column (Nickel beads Cat.1018; ADAR Biotech, USA). The purified protein was eluted from the column with elution buffer (20 mM Tris-HCl (pH 8.0), 0.5 M NaCl, 255 mM imidazole) and underwent dialysis in 20 mM Tris-HCl buffer at pH 8.0 containing 10% glycerol. Finally, the dialysate was concentrated to 1 mg/ml.

**Multiplex ELISA**

For the measurement of cytokine levels in the splenocyte culture supernatants, splenocytes were isolated from immunized mice, seeded at 5 × 10^5^ cells/well in a 96-well plate, and stimulated with 1 μg/mL of SFTSV peptide mixture for 72 h at 37 °C. The concentrations of cytokines were detected using ELISA kits (Invitrogen; Thermo Fisher Scientific) according to the manufacturer’s instructions.

**Supplementary Figures**

**
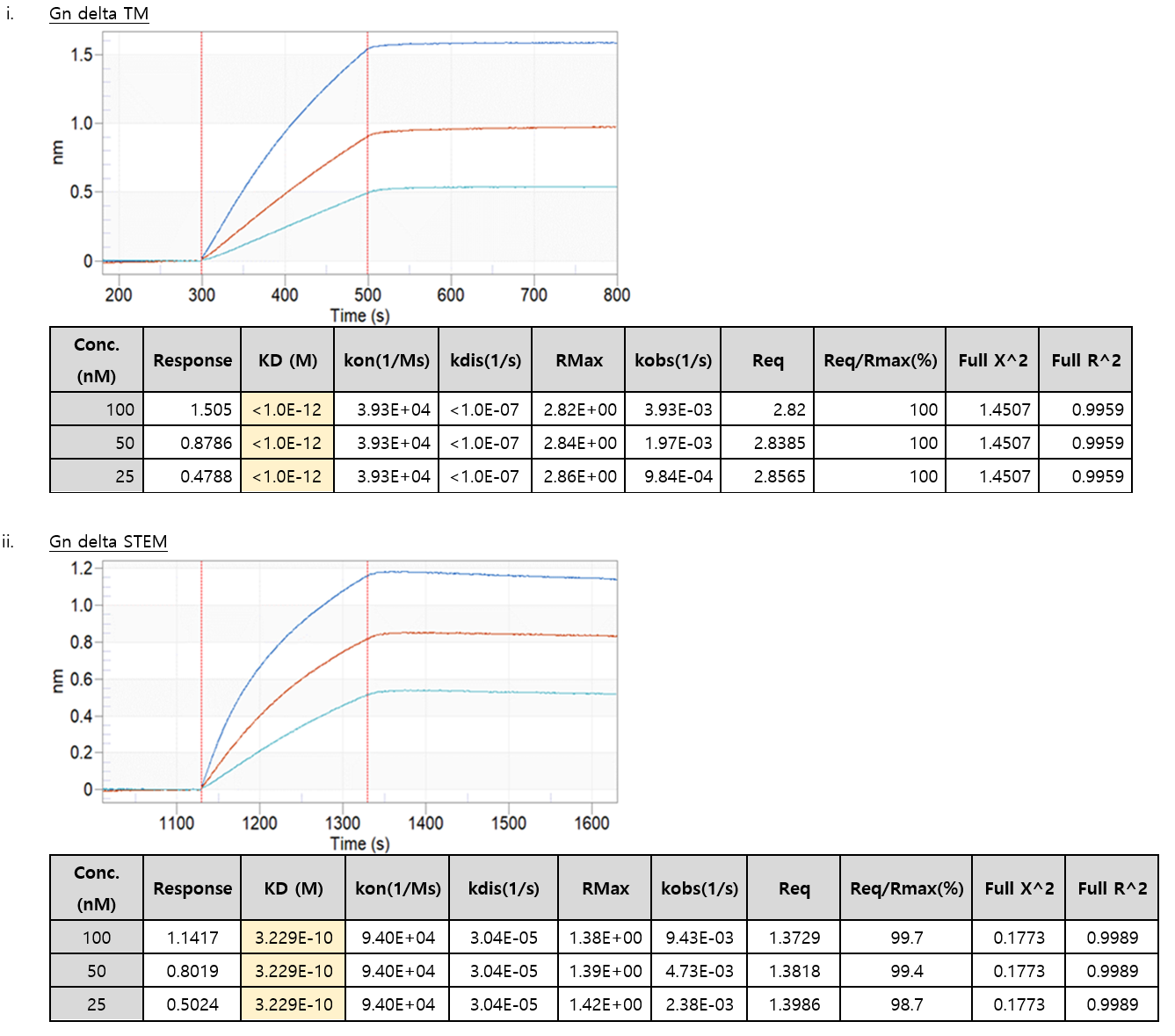
**

**Supplementary Figure 1. KD values of Ab10 antibody against GnΔTM and GnΔSTEM antigen.** The affinity of Ab10 towards immobilized SFTSV GnΔTM and GnΔSTEM was measured using biolayer interferometry.

**
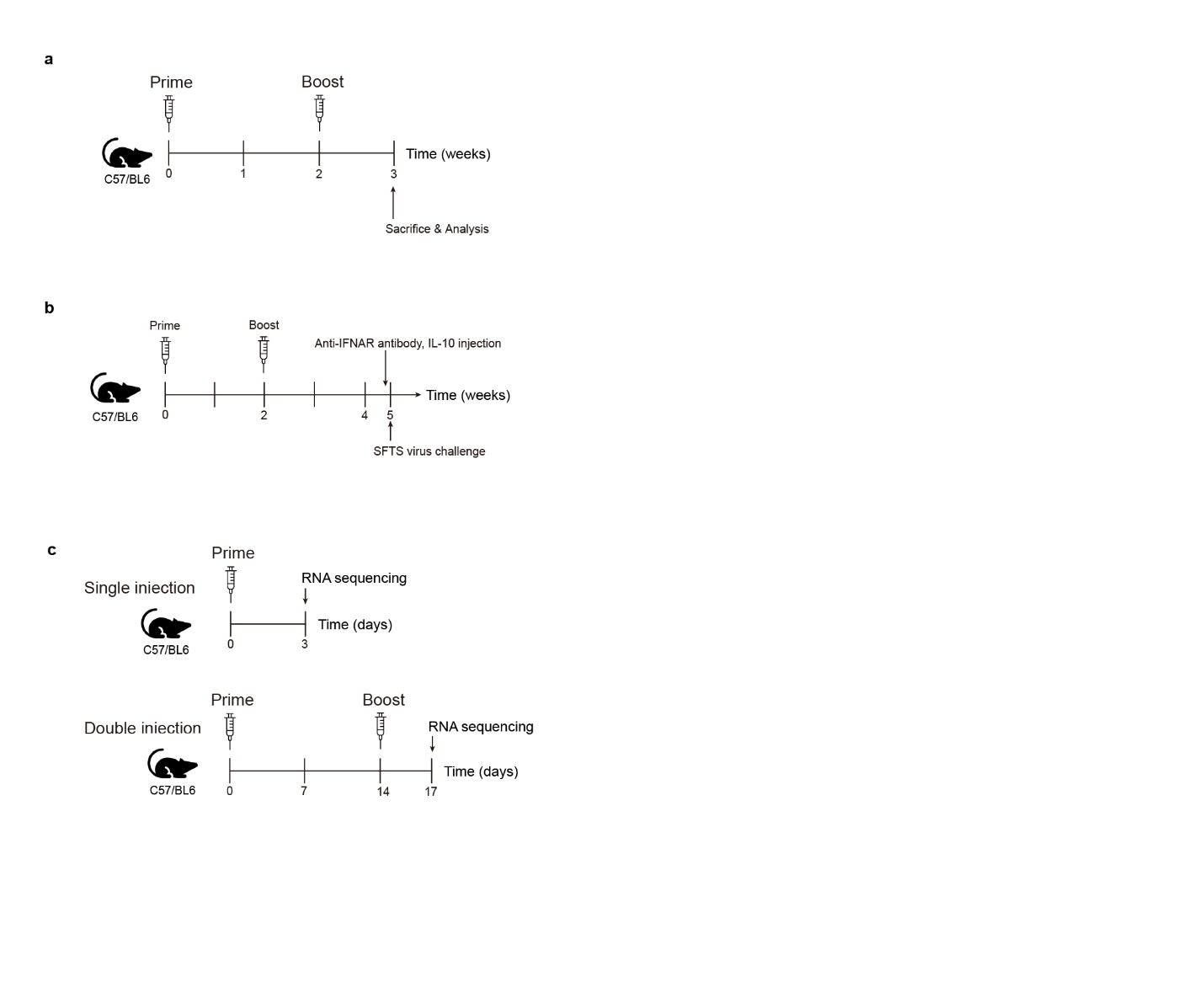
**

**Supplementary Figure 2. Immunization and virus challenge schedule of mice.** (a) C57/BL6 wildtype mice (n = 5/group) are immunized with rAd5-Gn (1 × 10^8^ IU) and/or Gn protein intramuscularly at 2-week intervals. One week after the boosting mice are sacrificed and analyzed. (b) C57/Bl6 wildtype mice (n = 5/group) are immunized with rAd5-Gn (1 × 10^8^ IU) and/or Gn protein intramuscularly at 2-week intervals. Mice are virus challenged 3 weeks after boosting. Mice receive the anti-interferon receptor antibody and IL-10 4 days and 1 day before virus challenge. (c) C57/BL6 wildtype mice (n = 4 or 5/group) are immunized with rAd5-Gn (1 × 10^8^ IU) and/or Gn protein. Single injection or double injection group are sacrificed 3 days after priming or boosting.

**
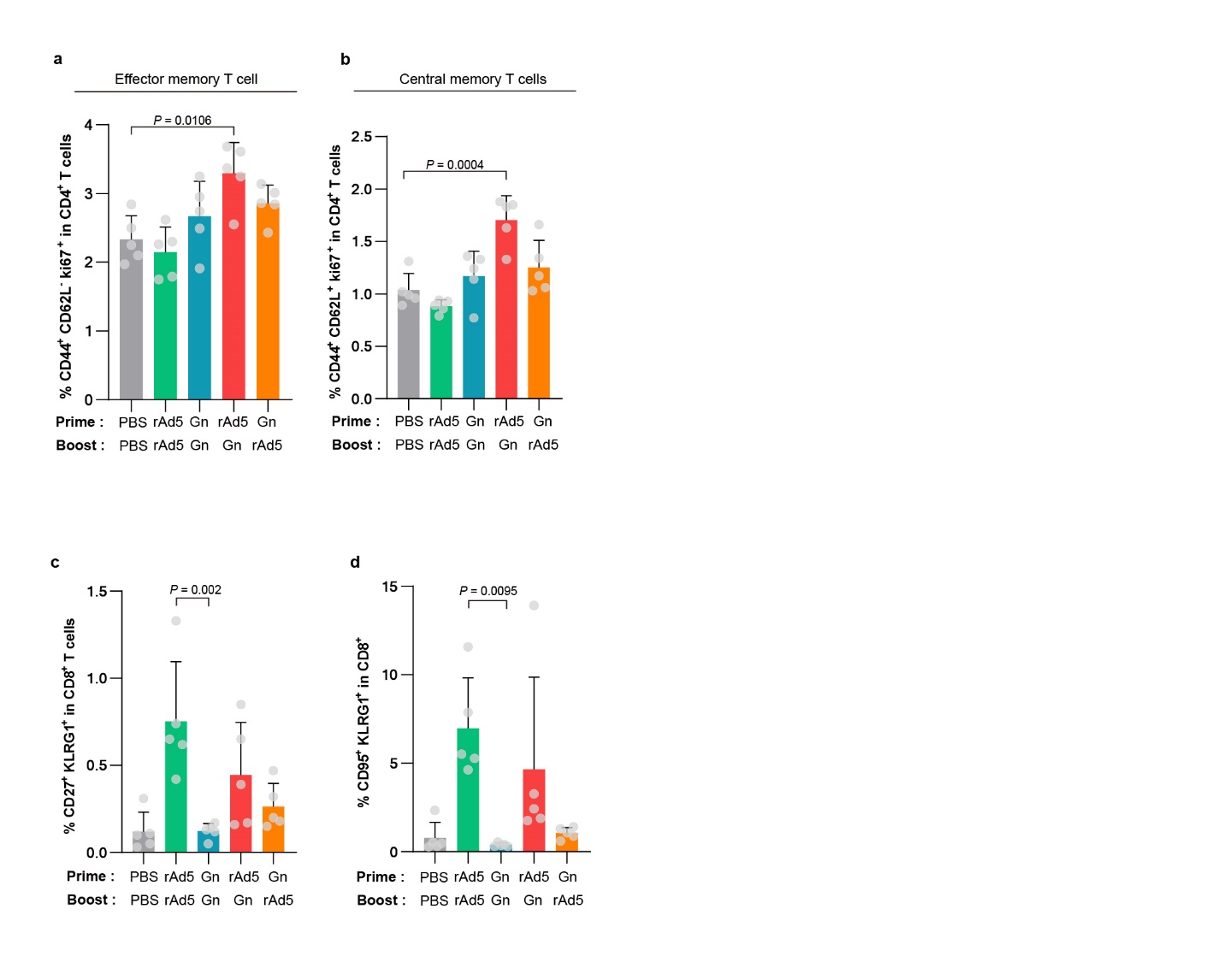
**

**Supplementary Figure 3. Analysis of T cell activation after heterologous or homologous immunization with the Ad5-Gn and Gn protein.** (a) C57/BL6 wildtype mice are immunized with rAd5-Gn (1 × 108 IU) and/or Gn protein intramuscularly at 2-week intervals and sacrificed 1 week after boosting. Splenocytes (5 × 105) from each mouse are cultured in the presence of Gn protein for 12 h. (b) Percentages of Ki-67+Tcm and Ki-67+Tem are assessed by flow cytometry. (c) The CD27+KLRG1+ population in CD8+ T cells in the spleen is assessed using flow cytometry. (d) The CD95+KLRG1+ population in CD8+ T cells in the spleen is assessed using flow cytometry. Data are presented as mean values ± SD. P-values are calculated using one-way ANOVA with Bonferroni multiple comparison test.

**
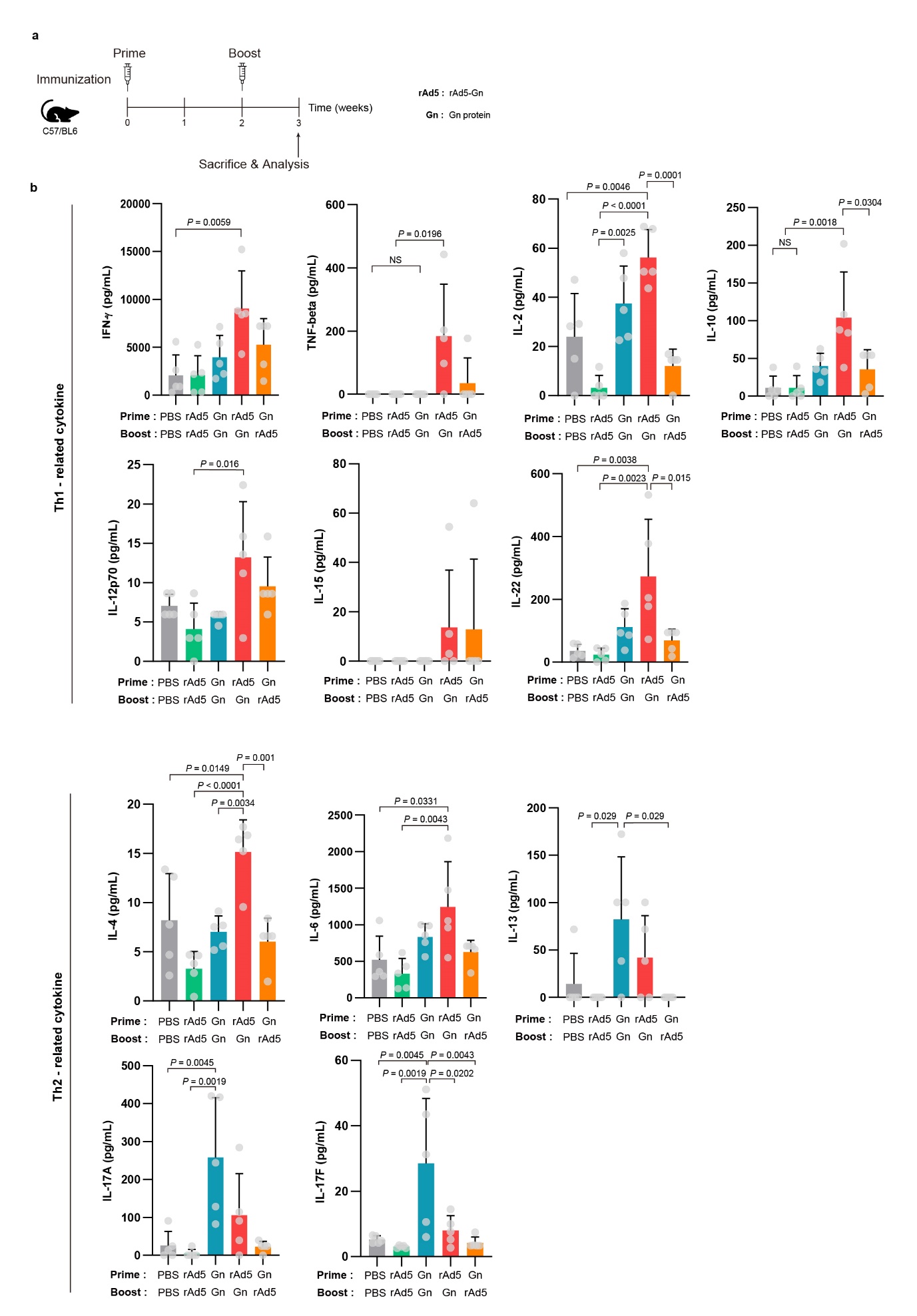
**

Supplementary Figure 4. Multiplex cytokine profiling of antigen-stimulated splenocytes from homologous or heterologous prime-boosted mice. (a) C57/BL6 wildtype mice are immunized with rAd5-Gn (1 × 10^8^ IU) and/or Gn protein intramuscularly at 2-week intervals and sacrificed 1 week after boosting. Splenocytes (5 × 10^5^) from each mouse are cultured in the presence of Gn protein for 3 days. (b) Cytokine concentrations in the cell culture supernatants are measured using multiplex analysis. Data are presented as mean values ± SDW. *P-*values are calculated using one-way ANOVA with Bonferroni multiple comparison test.


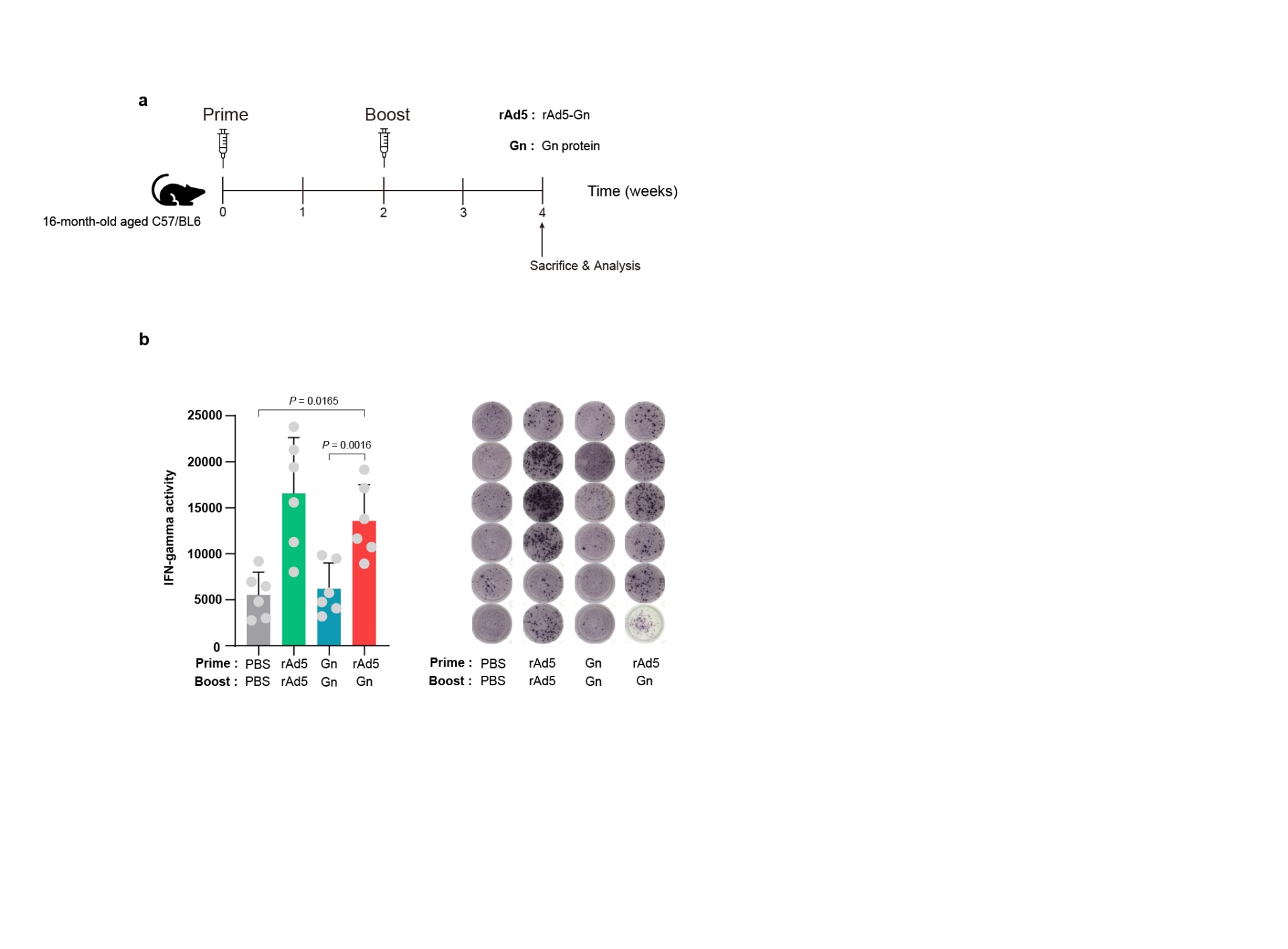


**Supplementary Figure 5. Analysis of IFN-γ-producing T cells after stimulation Gn peptide mix.** (a) 16-month-old C57/BL6 wildtype mice are immunized with rAd5-Gn (1 × 108 IU) and/or Gn protein intramuscularly at 2-week intervals. 1 week after boosting mice are sacrificed and analyzed. (b) Gn peptide mix-specific IFN-γ-producing T cells in the spleen were quantified using ELISPOT. Data are presented as mean values ± SD. P-values are calculated using one-way ANOVA with Bonferroni multiple comparison test.

**Follicular helper T cells (Figure 3b, c)**

**
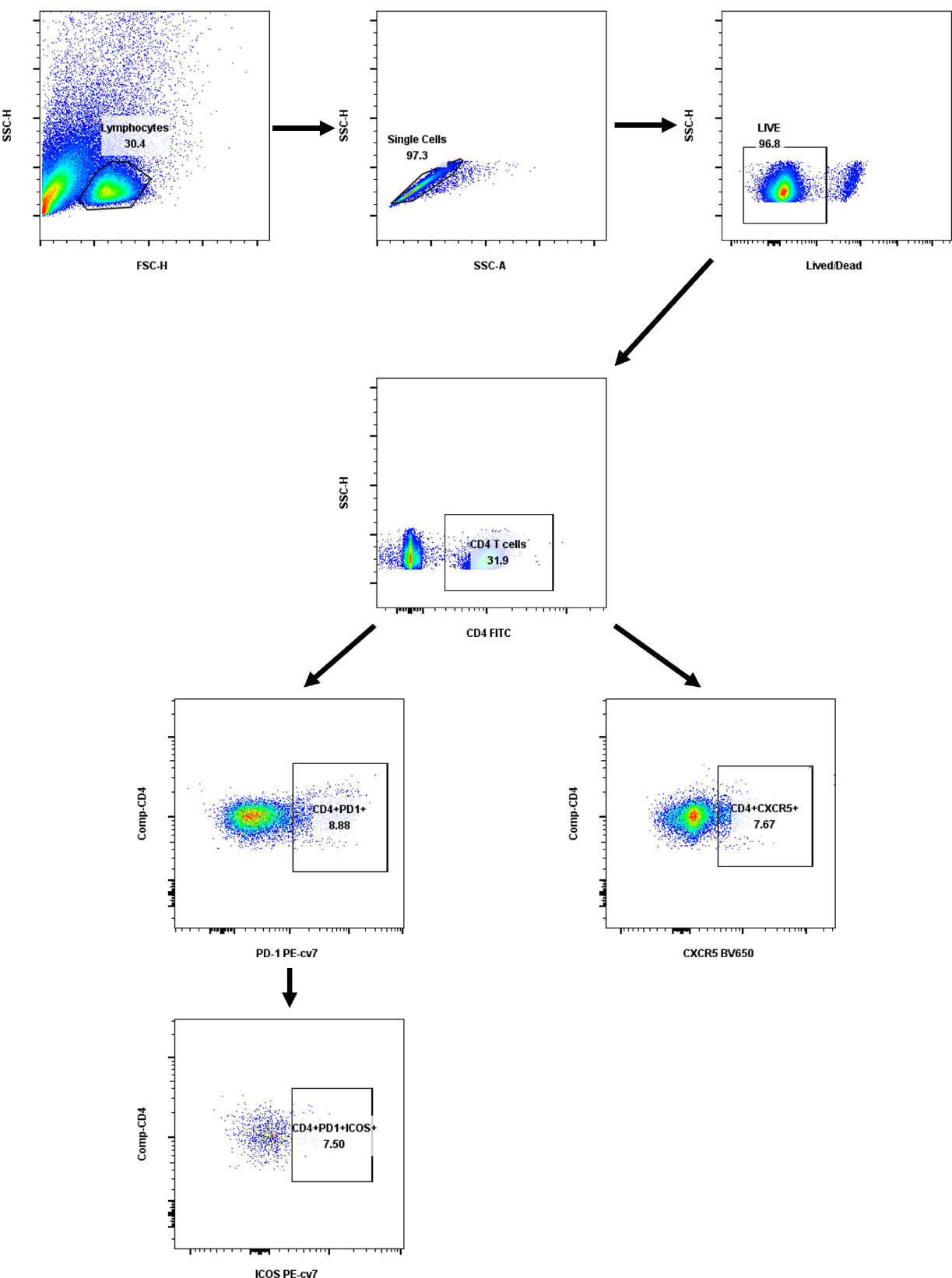
**

**Effector T cells and Central memory T cells (Figure 3e, f)**


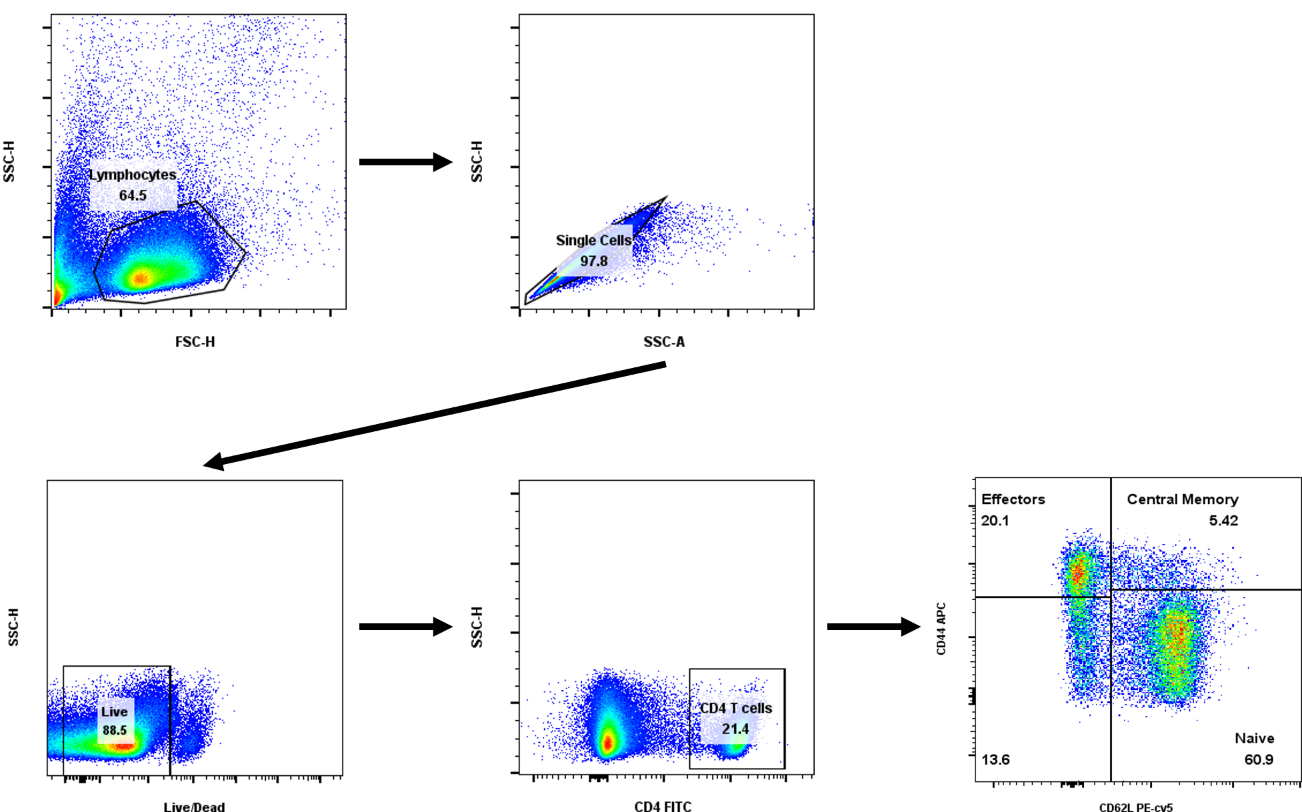


**CD4 and CD8^+^ IFN gamma (Figure 3h, i)**


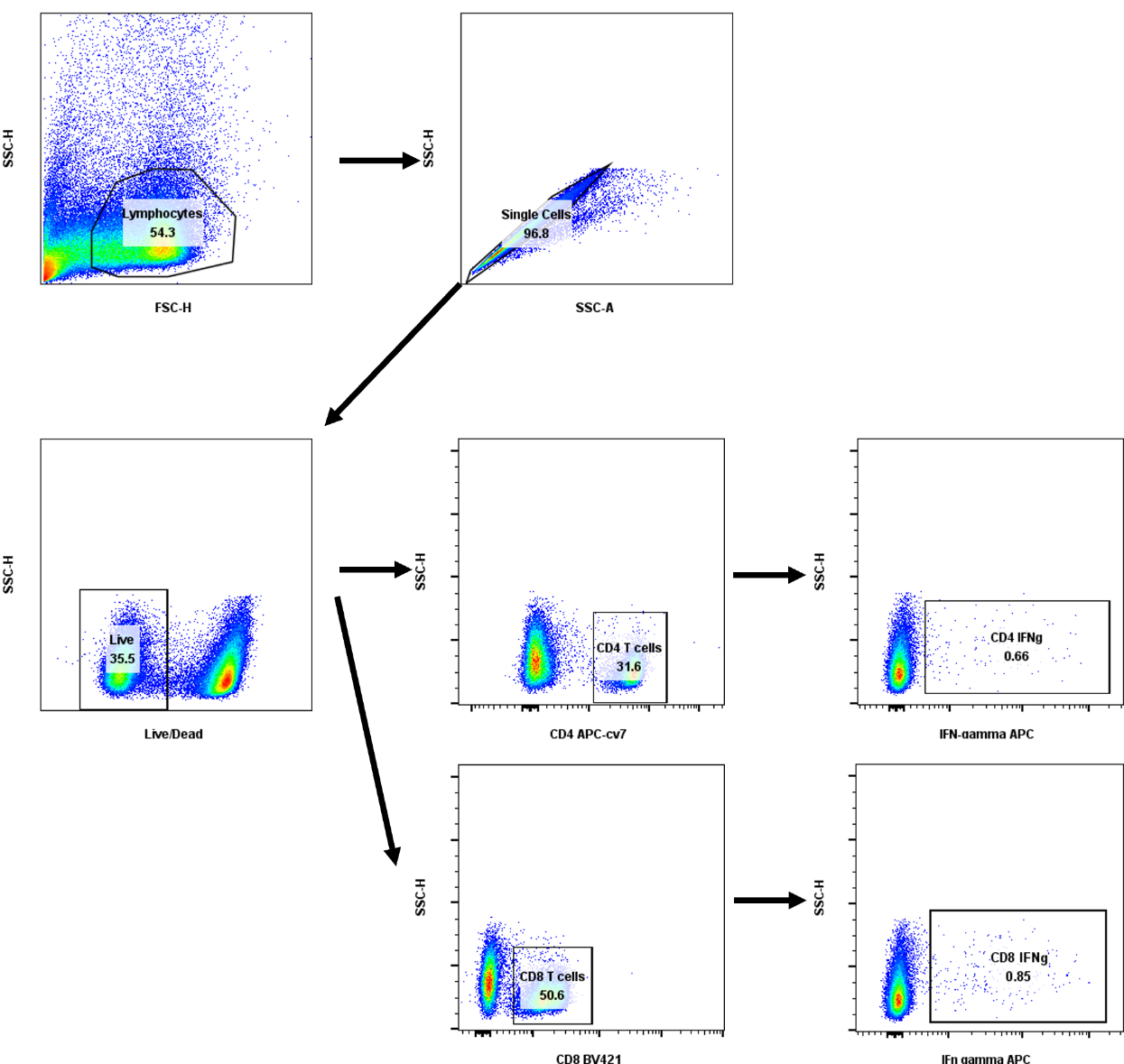


**T cell activation (Figure 3k, l)**


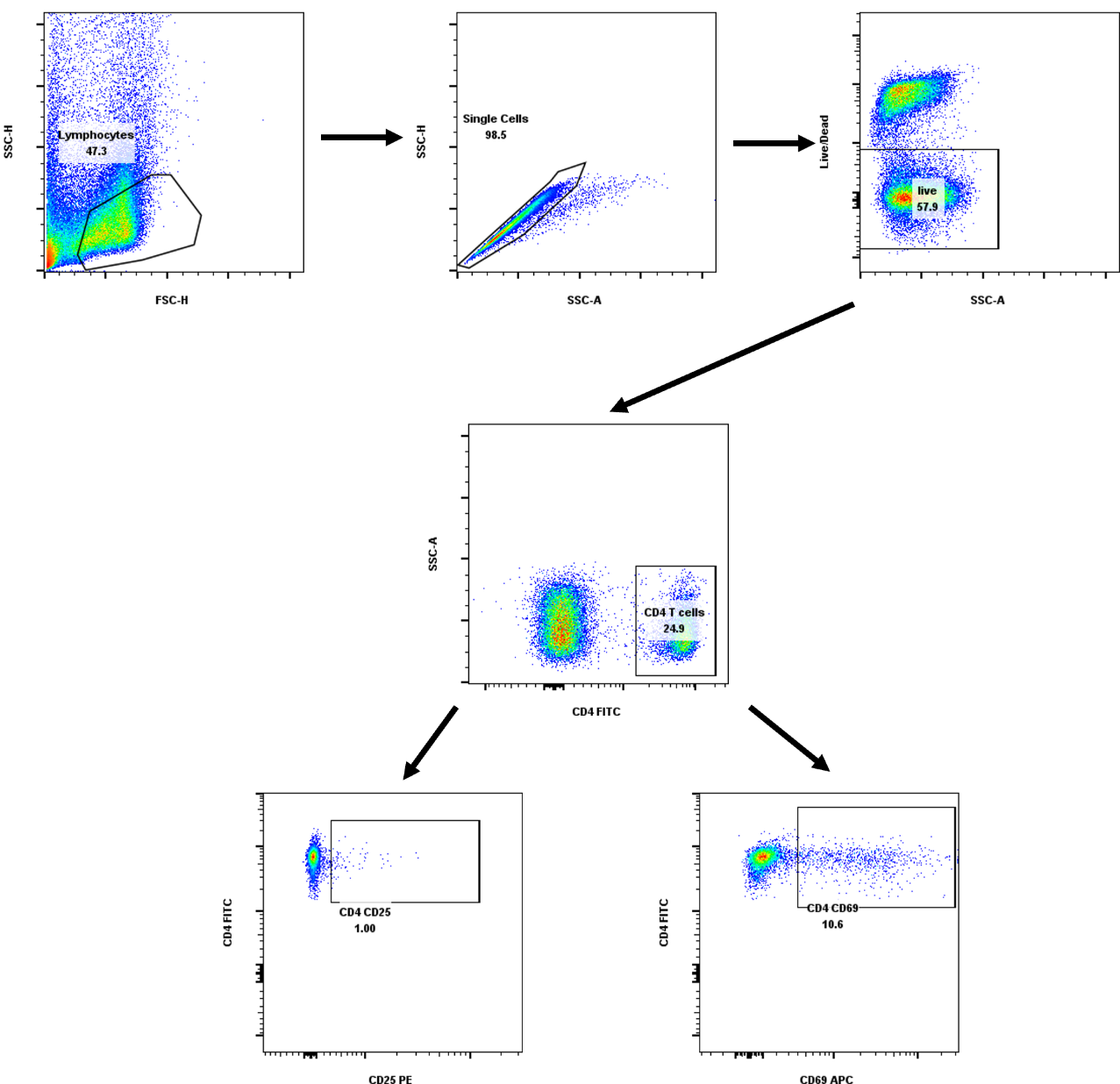


**Supplementary Figure 6. Flow cytometry gating strategy**
